# Retrospective exploratory study of smoking status and e‐cigarette use with response to non‐surgical periodontal therapy

**DOI:** 10.1002/JPER.21-0702

**Published:** 2022-08-16

**Authors:** Chandni Shah, Birte Holtfreter, Francis J. Hughes, Luigi Nibali

**Affiliations:** ^1^ Periodontology Unit Centre for Host Microbiome Interactions, Faculty of Dentistry Oral & Craniofacial Sciences King's College London London UK; ^2^ Department of Restorative Dentistry Periodontology Endodontology and Preventive and Pediatric Dentistry University Medicine Greifswald Greifswald Germany

**Keywords:** clinical study, periodontal debridement, periodontal diseases, smoking, vaping

## Abstract

**Background:**

To compare periodontal treatment responses in electronic cigarette (e‐cigarette) users, non‐smokers, former and current smokers.

**Methods:**

In this retrospective clinical study, 220 patients with periodontitis were seen for baseline periodontal charting, professional‐mechanical‐plaque‐removal (PMPR) and re‐evaluation by postgraduate students. Sixty of these patients were former smokers, twenty were former smokers now using e‐cigarettes, twenty current smokers, while all others (*n* = 120) were non‐smokers. Effects of smoking status and treatment duration on clinical outcomes were analyzed by linear models using generalized least squares adjusted for known confounders. The primary outcome was “need for surgery” defined as number of sextants with ≥2 non‐adjacent sites of probing depths (PD) ≥5 mm.

**Results:**

Compared with non‐smokers, e‐cigarette users had a less favorable treatment response after PMPR. This included statistically significant increased “need for surgery”, as well as increased number of sextants with PD ≥5 mm, number of sites with PD >5 mm and mean PD. There were no statistically significant differences between e‐cigarette users and current smokers. Former smokers responded statistically significantly better than e‐cigarette users for the primary outcome as well as for the number of sextants and sites with PD ≥5 mm and mean PD.

**Conclusions:**

Overall, e‐cigarette users had a statistically significantly less favorable response to PMPR than non‐smokers; their response was not statistically significantly different to those of current smokers. This, however, needs to be validated with further research in prospective clinical and observational studies in different populations.

## 1 INTRODUCTION

Many epidemiological and clinical studies have demonstrated the detrimental effects tobacco smoking has on the periodontium, making smoking a major risk factor for periodontitis.^1^ This effect is dose‐dependent, with increasing risk for heavy smokers compared with light smokers.^2^ The number of years of smoking cessation reduced the odds of periodontitis. Those who had quit smoking for ≥11 years had the same odds of having periodontitis as non‐smokers.^3^

The exact mechanism of how cigarettes affect the periodontium is not fully understood but there are several suggested mechanisms, such as an impaired immune and inflammatory response, effect on the oral bacteria and healing capacity.^4^, ^5^ These can lead to increased bone loss, attachment loss, deeper pockets (especially anterior and maxillary palatal sites), fibrotic gingiva, reduced gingival erythema, reduced edema, reduced bleeding on probing (BOP) and increased tooth loss.^6^ Most studies suggest that smokers can show improvement after PMPR (professional‐mechanical‐plaque‐removal) by reduction in PD (probing depth) but their response of PD reduction and clinical attachment gain is significantly less than in non‐smokers.^1^, ^7^, ^8^

Smoking cessation can improve the overall success of periodontal treatment outcomes and so this should be advised to patients.^9^ One of the most recent suggested methods for smoking cessation is electronic cigarettes (also termed “e‐cigarettes” or “vaping”). E‐cigarettes have been introduced to the UK since 2007 and their use among smokers and recent former smokers has continued to rise.^10^ E‐cigarettes provide nicotine in a vapor form for inhalation by heating a solution containing water, propylene glycol, and vegetable glycerin.

There has been research investigating the effects of e‐cigarettes to oral health.^11^, ^12^ It has been indicated that once smokers switched to e‐cigarettes, their BOP and gingival crevicular fluid (GCF) increased, suggesting restoration of the normal inflammatory response to plaque, that may be suppressed when smoking cigarettes.^13^ However, in vitro studies demonstrated that e‐cigarette vapor can lead to hyperinflammatory responses, alteration of human gingival epithelial cell morphology, apoptosis, and necrosis.^14^, ^15^ Data from Korea National Health and Nutrition Examination Survey showed similar odds of periodontitis for e‐cigarette and cigarette users, both having higher odds than non‐smokers.^16^, ^17^ However, it is important to recognize that e‐cigarette users were likely to be recent former smokers or dual users (also using cigarettes), and this could be contributing to the observations seen, as this was not accounted for. This can make this group of patients challenging to interpret. A systematic review concluded that e‐cigarettes may increase the risk of periodontitis and components of the e‐cigarette vapor could be carcinogenic, cytotoxic, and genotoxic, but more studies are needed to investigate this further.^18^ A feasibility study by Holliday et al. (2019) assessed the viability of delivering and evaluating e‐cigarettes as an intervention for smoking cessation. The patients in the two groups (smokers and e‐cigarettes) also received non‐surgical therapy. However, due to the nature of the study, the treatment outcomes between the groups were not assessed statistically and therefore we cannot compare their differences in treatment responses.^19^

There are currently no clinical studies evaluating the periodontal treatment outcomes in e‐cigarette users in comparison to non‐smokers, former smokers, and current smokers. Therefore, we aimed to investigate this response in these group of patients. We hypothesized that e‐cigarette users respond to PMPR in the same way as non‐smokers.

## 2 MATERIALS AND METHODS

### 2.1 Study design

This was a retrospective study over 24 months, where consecutive records of patients treated by postgraduate periodontology students at Guy's Dental Hospital were analyzed (screened from April 2018 to April 2020). Patients fulfilling the pre‐set inclusion criteria had their periodontal indices recorded pre‐ and post‐PMPR (measured ≥6 weeks post‐PMPR).

PMPR consisted of supra‐ and sub‐gingival mechanical plaque and calculus removal (under local anesthesia in PD > 4 mm). Initial treatment also involved oral health evaluation and education (toothbrushing and interdental cleaning advice). Primary diseases, for example, caries, were also addressed. Instruments used were a combination of ultrasonic scalers and hand curettes. The time spent instrumenting per session was between 60 and 90 min and was undertaken as full, half or quarter‐mouth sessions. Where the debridement was over multiple appointments, the time between each PMPR session ranged from 1 to 4 weeks.

Anonymized patient records were obtained without the need for individual patient consent. The study was approved as a service evaluation by the Quality Improvement and Patient Safety manager, Guys and St Thomas’ NHS Trust (project number: 11425, October 2020). It was conducted in accordance with the Helsinki Declaration of 1975, as revised in 2013.

### 2.2 Participants

A preliminary audit of patients attending the periodontology postgraduate clinic in the past 4 years, identified 20 current smokers and 20 e‐cigarette users, whose records were eligible for inclusion in the study. To calculate the sample size for this study we used these numbers and included consecutive patients that were former smokers and non‐smokers. In the absence of any data on e‐cigarette use and outcomes, response rates in terms of “closed pockets” for non‐smokers and e‐cigarette users (effect size) were estimated as 78% and 47%, respectively, based on previous studies on the effect of smoking.^20^

These figures were entered into an online Chi‐squared test calculator (http://hedwig.mgh.harvard.edu/sample_size/fisher/js/fisher.html), demonstrating that the study would have a power of 80% to detect a difference between non‐smoking groups with α = 0.05.

The patients were analyzed according to four study groups depending on their smoking status: non‐smokers, former smokers, current smokers, and e‐cigarette users. All records were screened consecutively for each group. Patients that met the inclusion criteria had a coded number recorded for inclusion in the study.

### 2.3 Inclusion/exclusion criteria

Inclusion criteria were (i) periodontitis diagnosis (localized/generalized, all stages and grades)^21^, (ii) adults (≥18‐years‐old), (iii) received PMPR by a periodontology postgraduate, and (iv) periodontal indices measured and recorded pre‐PMPR and 6–20 weeks post‐PMPR.

Exclusion Criteria were (i) uncontrolled diabetes (HbA1c > 7.5% or 58 mmol/mol), (ii) medications that could contribute to drug induced overgrowth (cyclosporin, amlodipine or phenytoin), (iii) pregnancy, (iv) any other forms of periodontal disease (other than periodontitis), (v) previous surgical treatment of periodontitis, (vi) use of adjunctive therapy to PMPR (systemic/local antimicrobials, immunomodulating treatment), and (vii) patients reporting concomitant use of e‐cigarettes and cigarettes (dual users).

### 2.4 Definitions of smoking status

The definitions below were used^3^, ^22^:

Former smokers were defined as anyone who had not smoked for > 1 year up to a maximum of 10 years and previously smoked ≥100 cigarettes (anyone who had quit smoking recently, e.g., < 1 year was excluded from the study).

Current cigarette smokers were defined as smoking ≥1 cigarette(s) a day, of ≥100 cigarettes in total.

E‐cigarette users were defined as former smokers that had quit smoking and using only e‐cigarettes of any form (not smoking cigarettes).

Non‐smokers were defined as anyone who had never used any form of cigarettes/e‐cigarettes within 10 years of baseline records to re‐evaluation records.

The groupings of each patient were based on patient self‐reporting.

### 2.5 Data collection

Baseline demographic, baseline periodontal clinical data, number of PMPR visits, and treatment duration (number of months from first to last PMPR visit) were collected from patient records. Post‐PMPR periodontal clinical data were also collected. Compliance was measured as attending or not attending appointments and were categorized into being compliant (> 90% of appointments attended) or non‐complaint (≤10% of appointments attended).

### 2.6 Outcome measures

The primary outcome was “need for surgery”, defined as the number of sextants with ≥2 non‐adjacent sites with PD ≥5 mm post‐PMPR. The European Federation of Periodontology clinical practice guidelines suggest that residual pockets can lead to further disease progression and tooth loss, therefore further treatment is recommended for these sites.^23^, ^24^ Secondary outcomes were number of sextants with ≥1 site with PD ≥5 mm, along with conventional measures of periodontitis severity, namely, mean PD, mean clinical attachment level (CAL), mean recession, full‐mouth‐bleeding‐score (FMBS), and full‐mouth‐plaque‐score (FMPS).

All anonymized data were entered into a dedicated encrypted database by the same periodontology postgraduate (author C.S.). Approximately 10% of the data were entered in duplicate, by a different colleague, to check for accuracy, demonstrating 100% accuracy. Access was restricted to data entry personnel and study investigators. Upon completion, the data were proofed for entry errors.

### 2.7 Statistical analysis

Continuous data were presented as means and standard deviations, together with median, 25% and 75% quantiles. Categorical data were presented as numbers and percentages. To check for distributional differences across the four groups, Kruskal‒Wallis tests and Chi‐squared tests were applied as appropriate. Violin plots were used to visualize the data, containing boxplots and estimated distributions.

**FIGURE 1:**
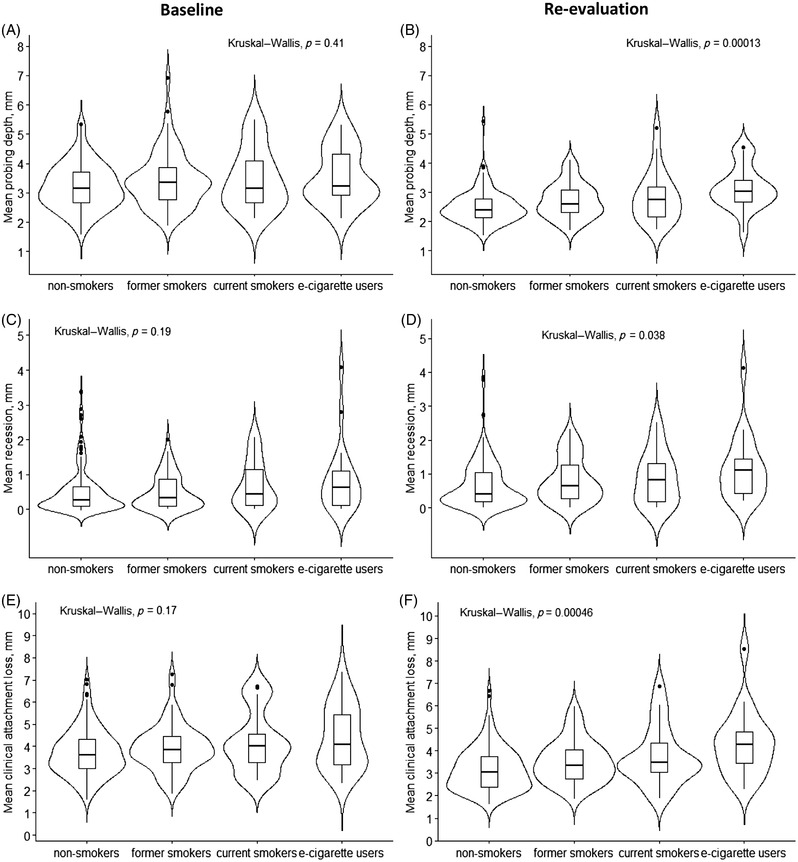
Baseline (left) and re‐evaluation (right) levels of mean probing depth (**A, B**), mean recession (**C, D**) and mean clinical attachment loss (**E, F**) by smoking status. *P* values from Kruskal‒Wallis tests are provided

**FIGURE 2:**
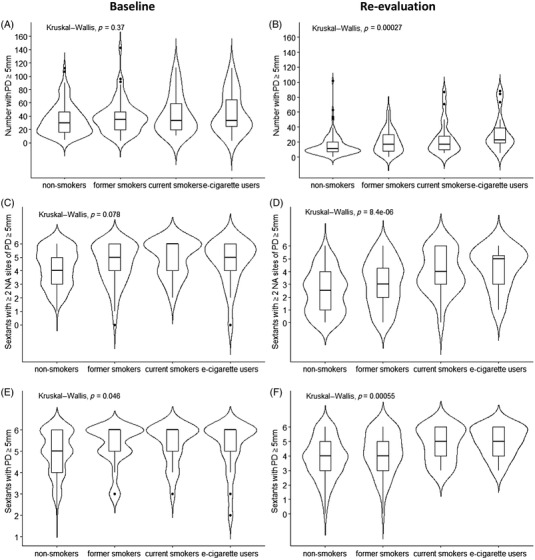
Baseline (left) and re‐evaluation (right) levels of the number with probing depths (PD) ≥5 mm (**A, B**), the number of sextants with ≥2 non‐adjacent (NA) sites with PD ≥5 mm (**C, D**), and the number of sextants with PD ≥5 mm (**E, F**) by smoking status. *P* values from Kruskal‒Wallis tests are provided

Fully adjusted regression models were evaluated to estimate effects of smoking status on periodontal outcomes, including interaction terms with treatment duration (see Figures 1 and 2, Tables S2‒S11 for models and Table S12 for contrast estimates in online *Journal of Periodontology*). Linear models using generalized least squares were applied to evaluate effects of smoking, treatment duration (in exact months), and their interaction on periodontal outcomes. Models were adjusted for age, sex, compliance, number of PMPR sessions, and any medical conditions. We did not adjust for baseline outcome status, as this was assumed to be a mediator of the effect of smoking on follow‐up outcome status, as smoking already affected the baseline outcome status.^25^ All continuous variables were modeled using restricted cubic splines (identified as RCS1 and RCS2) with three knots to allow for non‐linearity. Linear regression coefficients (B), 95% confidence intervals (CI) and *P* values were reported (see Figure S1‒S7 and Tables S1‒S13 in online *Journal of Periodontology*). Based on the estimated regression coefficients, smoking contrasts (as beta coefficients with 95% CIs) contrasting e‐cigarette users, current smokers, and former smokers with non‐smokers were calculated and graphically visualized. Additionally, smoking contrasts for the comparison of effects (beta coefficients with 95% CIs) in e‐cigarette users versus current smokers were calculated, tabulated, and graphically presented.

All regression models were re‐run restricting patients to former smokers and e‐cigarette users, adjusting also for the number of years since patients quitted smoking. Contrasts (corresponding to beta coefficients with 95% CIs for respective comparisons) were tabulated and graphically presented.

*P* values <0.05 were considered statistically significant. Analyses were conducted using various statistical analysis software.* ^,^ †

## 3 RESULTS

### 3.1 Baseline characteristics

The study sample in all four groups were treated and assessed before and after periodontal treatment by 13 operators. Each operator treated a mixed range of all four groups (see Table S1 in online *Journal of Periodontology*).

A total of 220 subjects were included (Table 1); 120 non‐smokers, 60 former smokers, 20 current smokers, and 20 e‐cigarette users. The ages of the population ranged from 18 to 77 years. The ethnicity of the subjects could not be analyzed as only 31% were recorded. No statistically significant differences for diagnosis staging and grading across the groups were detected. The patients included in the study did not have previous periodontal therapy before the data were collected. The treatment duration in months had statistically significant differences amongst the smoking groups (*p* < 0.01). The e‐cigarette users had the longest treatment duration. There were no significant effects for time between PMPR and re‐assessment (see Table S13 in online *Journal of Periodontology*). The most compliant groups were non‐smokers and former smokers, while e‐cigarette users and current smokers had similar compliance.

**TABLE 1:** Baseline characteristics in total and stratified by smoking status (*N* = 220)

Baseline characteristics	Total (*N* = 220)	Non‐smokers (*n* = 120)	Former smokers (*n* = 60)	Current smokers (*n* = 20)	E‐cigarette users (*n* = 20)	*p* value^a^
Time from baseline date to re‐evaluation appointment, weeks	28.7±14.7 26 (18; 35)	25.9 ± 13.5 23 (16; 31)	30.3±14.4 27 (19; 41)	30.0±15.8 26 (22; 33)	39.9±16.2 39 (27; 50)	**0.0006**
Time from baseline date to first PMPR appointment, weeks	14.5±13.1 11 (5; 20)	12.4±12.0 9 (4; 17)	15.2±12.1 11 (7; 24)	15.3±14.8 13 (7; 18)	23.8±16.7 24 (11; 35)	**0.009**
Time from first to last PMPR appointment, weeks	3.2±2.6 3 (1; 5)	2.6±2.3 2 (1; 4)	4.2±2.7 4 (2; 7)	3.8±2.8 5 (1; 6)	3.5±2.7 3 (1; 5)	**0.002**
Time from last PMPR appointment to re‐evaluation appointment, weeks	11.0±2.9 11 (9; 13)	10.8±2.9 10 (9; 13)	10.9±3.0 10 (8; 14)	10.9±2.5 11 (9; 13)	12.6±3.0 13 (11; 15)	**0.0005**
Number of PMPR sessions	2.2±0.8 2 (2; 2)	2.1±0.8 2 (2; 2)	2.4±0.9 2 (2; 3)	2.0±0.8 2 (1.5; 2)	2.2±0.9 2 (2; 3)	0.14
Age, years	47.9±11.1 48 (41; 55)	48.0±12.2 48 (40.5; 57)	49.0±9.8 49 (44; 54)	44.1±10.2 43 (34.5; 53)	47.8±6.9 49 (43.5; 54)	0.39
Male sex	90 (40.9%)	48 (40.0%)	22 (36.7%)	11 (55.0%)	9 (45.0%)	0.52
Number of cigarettes smoked	NA	NA	NA	10.2±6.4 8 (5; 17.5)	NA	NA
Years smoked	NA	NA	NA	20.0±9.9 20 (14.5; 25.5)	NA	NA
Years since quitting smoking	NA	NA	4.9±3.7 3 (1.8; 9)	NA	3.4±2.6 2 (1.5; 5) ^b^	NA
Pack‐years	NA	NA	NA	10.7±8.5 9.2 (3.4; 17.5)	NA	NA
Any medical conditions, yes	75 (34.1%)	33 (27.5%)	25 (41.7%)	7 (35.0%)	10 (50.0%)	0.11
Diagnosis extent						
Generalized	171 (77.7%)	93 (77.5%)	46 (76.7%)	14 (70.0%)	18 (90.0%)	
Localized	49 (22.3%)	27 (22.5%)	14 (23.3%)	6 (30.0%)	2 (10.0%)	0.48
Diagnosis stage						
II	4 (1.8%)	4 (3.3%)	0 (0%)	0 (0%)	0 (0%)	
III	43 (19.6%)	22 (18.3%)	15 (25.0%)	3 (15.0%)	3 (15.0%)	
IV	173 (78.6%)	94 (78.3%)	45 (75.0%)	17 (85.0%)	17 (85.0%)	0.54
Diagnosis grade						
B	9 (4.1%)	5 (4.2%)	3 (5.0%)	1 (5.0%)	0 (0%)	
C	211 (95.9%)	115 (95.8%)	57 (95.0%)	19 (95.0%)	20 (100.0%)	0.80
Compliance						
No	65 (29.6%)	27 (22.5%)	20 (33.3%)	9 (45.0%)	9 (45.0%)	
Yes	155 (70.5%)	93 (77.5%)	40 (66.7%)	11 (55.0%)	11 (55.0%)	**0.049**

Data are reported as means and SDs, along with medians, 25% and 75% quantiles.

Findings with statistical significance, *p* < 0.05, have been formatted in bold.

Abbreviation: PMPR, professional mechanical plaque removal.

^a^ Chi‐squared or Kruskal‒Wallis test.

^b^ *n* = 19.

The current smoker group smoked a mean of 10.2 cigarettes daily (10.7 pack‐years). The e‐cigarette group used e‐cigarettes for a mean of 2.4 years. The e‐cigarette users had quit smoking cigarettes for an average of 3.3 years.

### 3.2 Periodontal status at baseline and re‐evaluation

Most periodontal parameters at baseline were similar amongst all groups with any differences being not statistically significant, except for number of sextants with PD ≥5 mm (*p* = 0.046). At re‐evaluation, the number of teeth (excluding wisdom teeth), FMBS, and FMPS were not significantly different amongst the groups. However, when considering the primary outcome (“need for surgery” or number of sextants with ≥2 non‐adjacent sites of PD ≥5 mm) the groups did not respond in the same way to periodontal treatment (*p* <0.01), resulting in e‐cigarette users “needing” a mean of 4.3 surgeries, compared with 4 for current smokers, 3.1 for former smokers, and 2.4 for non‐smokers.

**FIGURE 3:**
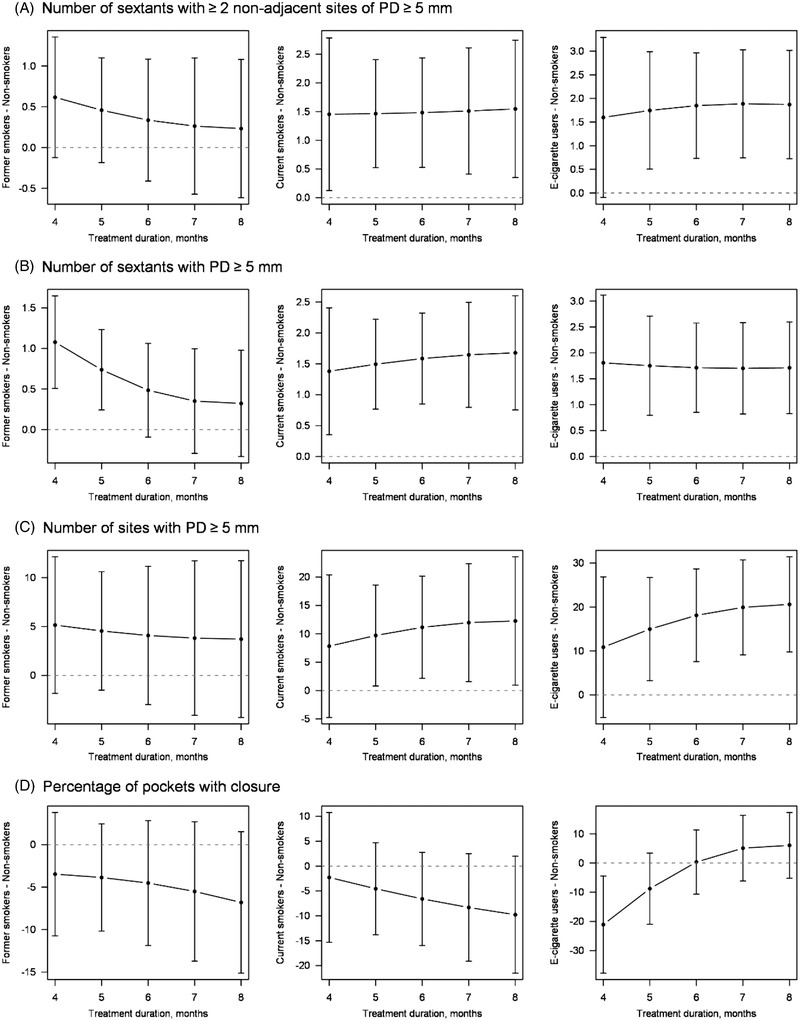
Predicted contrasts (from left to right: former smokers, current smokers, e‐cigarette users; see Table S12) from linear models (see Tables [Link], [Link], [Link], [Link]) analyzing effects of smoking on (**A**) the number of sextants with ≥2 non‐adjacent sites of probing depth (PD) ≥5 mm, (**B**) the number of sextants with pocket probing depth ≥5 mm, (**C**) the number of sites with pocket probing depths ≥5 mm, and (**D**) the percentage of pockets with closure. Predicted contrasts with 95% confidence intervals are shown. The horizontal dashed line equals a contrast of zero (i.e., no difference)

**FIGURE 4:**
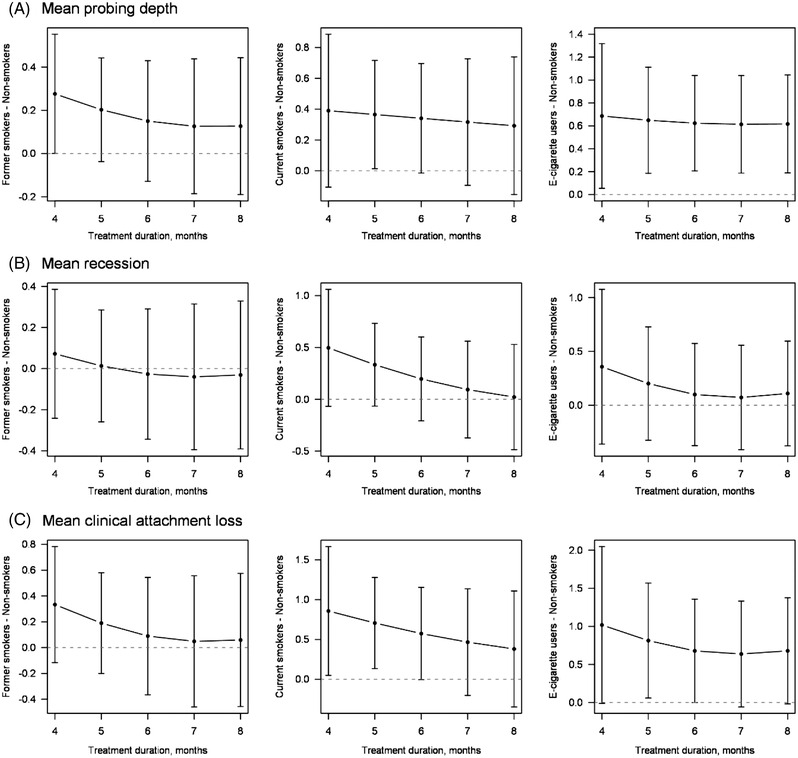
Predicted contrasts (from left to right: former smokers, current smokers, e‐cigarette users; see Table S12) from linear models (see Tables [Link], [Link], [Link]) analyzing effects of smoking on (**A**) mean probing depth, (**B**) mean recession, and (**C**) mean clinical attachment loss. Predicted contrasts with 95% confidence intervals are shown. The horizontal dashed line equals a contrast of zero (i.e., no difference)

E‐cigarette users also had the least favorable treatment response when considering the secondary outcome measures; mean PD (*p* <0.01), mean recession (*p* = 0.038), mean CAL (*p* <0.01), and number of sextants with PD ≥5 mm (*p* < 0.01). Violin plots were used to help visualize the data (Figures 3 and 4).

### 3.3 Effects of smoking status on periodontal treatment outcomes

Pocket closure (sites with PD ≤4 mm without BOP after treatment) occurred on average in 77.1% of sites in non‐smokers, 74.9% of sites in former smokers, 69.4% of sites in current smokers, and 66.6% of sites in e‐cigarette users (Table 2).

**TABLE 2:** Periodontal data at baseline and re‐evaluation in total and stratified by smoking status

**Periodontal data**	**Total (*N* = 220)**	**Non‐smokers (*n* = 120)**	**Former smokers (n = 60)**	**Current smokers (*n* = 20)**	**E‐cigarette users (*n* = 20)**	***p* value** *
Number of sextants with ≥2 non‐adjacent sites of PD ≥5 mm						
Baseline	4.4±1.4 5 (3; 6)	4.3±1.4 4 (3; 5)	4.5±1.5 5 (4; 6)	5.0±1.3 6 (4; 6)	4.7±1.6 5 (4; 6)	0.08
Re‐evaluation	2.9±1.8 3 (1; 4)	2.4±1.7 2.5 (1; 4)	3.1±1.9 3 (2; 4.5)	4.0±1.7 4 (3; 6)	4.3±1.6 5 (3; 5.5)	0.0001
Change	‐1.5±1.4 ‐1 (‐2; 0)	‐1.9±1.4 ‐2 (‐3; ‐1)	‐1.5±1.4 ‐1 (‐2.5; 0)	‐1.0±1.4 0 (‐2; 0)	‐0.4±0.8 0 (‐1; 0)	0.0001
Number of teeth (excluding wisdom teeth)						
Baseline	25.7±2.6 26 (24; 28)	26.0±2.5 27 (25; 28)	25.4±2.7 26 (24; 27.5)	25.4±2.2 25 (23.5; 28)	25.4±3.2 26 (23.5; 28)	0.16
Re‐evaluation	25.5±2.7 26 (24; 28)	25.9±2.6 27 (24; 28)	25.0±2.8 25 (24; 27)	25.1±2.6 25 (23.5; 28)	25.1±3.4 26 (23.5; 28)	0.09
Change	0.2±0.7 0 (0; 0)	0.1±0.4 0 (0; 0)	0.4±1.1 0 (0; 0)	0.3±0.7 0 (0; 0)	0.3±0.6 0 (0; 0)	0.48
Full‐mouth plaque score, %						
Baseline	47.5±20.1 47.5 (31; 63)	46.3±19.1 47 (31; 60.5)	49.6±21.2 49.5 (34.5; 66)	47.8±21.6 46 (27; 66.5)	48.6±21.4 53.5 (30; 62.5)	0.86
Re‐evaluation	22.5±15.3 20 (11; 29)	20.3±12.3 19 (11; 25.5)	24.8±17.3 23 (10.5; 31)	23.5±12.2 21.5 (12.5; 31)	27.9±24.0 18.5 (11; 41.5)	0.40
Change	‐25±19.6 ‐23 (‐38; ‐11)	‐26.0±19.9 ‐26 (‐39; ‐9)	‐24.7±17.6 ‐21 (‐35.5; ‐13)	‐24.3±20.8 ‐28 (‐40.5; ‐10.5)	‐20.8±23.2 ‐16.5 (‐34; ‐11)	0.88
Full‐mouth bleeding score, %						
Baseline	41.4±24.6 38 (20.5; 59.5)	41.2±23.1 38.5 (21.5; 57.5)	43.4±27.3 37.5 (20; 66)	43.8±23.6 44 (31.5; 60)	33.7±26.1 27.5 (12.5; 49)	0.35
Re‐evaluation	20.7±15.0 17 (10; 28.5)	20.1±13.8 17 (11; 25)	19.7±14.3 16.5 (8; 29)	22.6±18.4 19 (7; 31)	26.1±19.7 22.5 (10.5; 38)	0.72
Change	‐20.6±22.5 ‐17 (‐34; ‐6)	‐21.2±21.3 ‐16 (‐33.5; ‐6)	‐23.6±23.8 ‐20 (‐40.5; ‐7)	‐21.2±20.0 ‐21.5 (‐35.5; ‐4.5)	‐7.6±25.3 ‐7 (‐23.5; 9.5)	0.14
Number of sites with PD ≥5 mm						
Baseline	37.4±25.1 33 (17; 49.5)	34.6±23.2 29.5 (16; 47)	38.7±25.3 34.5 (19; 47.5)	42.5±29.8 33.5 (19.5; 61.5)	44.7±30.3 33 (24; 67)	0.37
Re‐evaluation	18.8±17.0 13 (8; 24.5)	14.9±13.7 11 (7; 20)	20.0±15.2 17 (8; 30)	25.0±22.6 17 (9.5; 29)	32.2±24.2 22.5 (17.5; 40)	0.0003
Change	‐18.6±18.8 ‐13 (‐27.5; ‐6)	‐19.8±18.4 ‐14 (‐28; ‐6.5)	‐18.6±16.6 ‐14.5 (‐27; ‐7)	‐17.5±22.6 ‐12.5 (‐33.5; ‐2)	‐12.5 ± 23.2 ‐8.5 (‐29.5; 1)	0.38
Mean PD, mm						
Baseline	3.33±0.87 3.21 (2.70; 3.81)	3.21±0.78 3.14 (2.65; 3.71)	3.44±0.95 3.36 (2.75; 3.90)	3.48±1.03 3.15 (2.61; 4.13)	3.53±0.95 3.23 (2.92; 4.34)	0.41
Re‐evaluation	2.65±0.65 2.54 (2.20; 2.97)	2.49±0.56 2.39 (2.13; 2.77)	2.72±0.59 2.60 (2.30; 3.08)	2.86±0.89 2.75 (2.15; 3.27)	3.15±0.74 3.02 (2.66; 3.49)	0.0001
Change	‐0.68±0.61 ‐0.61 (‐1.01; ‐0.30)	‐0.71±0.54 ‐0.61 (‐1.05; ‐0.34)	‐0.72±0.65 ‐0.66 (‐0.99; ‐0.29)	‐0.62±0.66 ‐0.58 (‐1.11; ‐0.21)	‐0.38±0.79 ‐0.42 (‐0.99; 0.28)	0.38
Mean recession, mm						
Baseline	0.56±0.67 0.33 (0.09; 0.81)	0.51±0.66 0.26 (0.08; 0.66)	0.48±0.50 0.32 (0.09; 0.86)	0.69±0.69 0.43 (0.11; 1.17)	0.88±1.01 0.62 (0.12; 1.16)	0.19
Re‐evaluation	0.78±0.73 0.60 (0.23; 1.19)	0.68±0.70 0.40 (0.19; 1.05)	0.81±0.66 0.64 (0.28; 1.27)	0.88±0.78 0.82 (0.19; 1.30)	1.17±0.95 1.12 (0.38; 1.55)	0.038
Change	0.22±0.46 0.13 (0; 0.45)	0.17±0.46 0.10 (‐0.01; 0.32)	0.32±0.44 0.18 (0.003; 0.63)	0.19±0.48 0.15 (‐0.004; 0.44)	0.28±0.44 0.19 (0.08; 0.45)	0.17
Mean CAL, mm						
Baseline	3.88±1.15 3.75 (3.12; 4.44)	3.72±1.07 3.63 (3.01; 4.34)	3.92±1.06 3.85 (3.27; 4.44)	4.17±1.40 4.01 (3.15; 4.76)	4.42±1.43 4.07 (3.18; 5.59)	0.17
Re‐evaluation	3.43±1.10 3.26 (2.58; 4.01)	3.18±0.98 3.04 (2.38; 3.74)	3.52±0.96 3.33 (2.74; 4.07)	3.74±1.30 3.47 (3.01; 4.46)	4.31±1.46 4.29 (3.33; 4.89)	0.0005
Change	‐0.46±0.67 ‐0.40 (‐0.84; ‐0.07)	‐0.55±0.62 ‐0.50 (‐0.89; ‐0.16)	‐0.40±0.66 ‐0.31 (‐0.74; ‐0.01)	‐0.43±0.73 ‐0.34 (‐0.90; 0.08)	‐0.10±0.88 ‐0.12 (‐0.75; 0.19)	0.094
Number of sextants with PD ≥5 mm						
Baseline	5.1±1.0 5 (5; 6)	5.0±1.0 5 (4; 6)	5.3±1.0 6 (5; 6)	5.4±0.9 6 (5; 6)	5.3 ± 1.2 6 (5; 6)	0.046
Re‐evaluation	4.1±1.4 4 (3; 5)	3.8±1.4 4 (3; 5)	4.2±1.5 4 (3; 5)	4.8±1.2 5 (4; 6)	5.0±1.0 5 (4; 6)	0.0005
Change	‐1.0±1.2 ‐1 (‐2; 0)	‐1.1±1.1 ‐1 (‐2; 0)	‐1.1±1.2 ‐1 (‐2; 0)	‐0.6±1.2 0 (‐1.5; 0)	‐0.3±0.6 0 (‐1; 0)	0.003
% pockets with closure						
Change	74.9 ±16.1 78.1 (64.7; 86.5)	77.1±14.2 80.5 (72.5; 86.7)	74.9±15.4 77.2 (63.4; 87.3)	69.4±20.9 74.9 (56.6; 85.9)	66.6±20.2 71.0 (48.1; 84.1)	0.11

Data are reported as means and SDs, along with medians, 25% and 75% quantiles.

*P values were derived from Kruskal‒Wallis tests.

Abbreviations: PD, probing depth; CAL, clinical attachment level.

Fully adjusted regression models including interaction terms with treatment duration, (see Figures 1 and 2, Tables S2‒S11 for models, Table S12 for contrast estimates in online *Journal of Periodontology*), showed that “need for surgery” at re‐evaluation was associated with smoking status. This showed significantly higher numbers of sextants with ≥2 non‐adjacent sites of PD ≥5 mm in the e‐cigarette users and the current smokers when comparing the treatment response to non‐smokers for treatment durations of 5‒8 months (Figure 1A). Effects were ≈ 1.5 sextants for former smokers and 1.7 for e‐cigarette users compared with non‐smokers.

Among secondary outcomes, the number of sextants with PD ≥5 mm (effects were ≈ 1.5 in current smokers and 1.7 in e‐cigarette users; Figure 1B), the number of sites with PD ≥5 mm (effects were ≈ 7‒12 in current smokers and 10‒21 in e‐cigarette users; Figure 1C), and mean PD (effects were ≈ 0.62 mm in e‐cigarette users; Figure 2A) differed significantly comparing current smokers or e‐cigarette users with non‐smokers. For mean CAL (Figure 2C), negative effects of current smoking were only seen between months 4 and 5, while negative effects of e‐cigarette usage were seen at month 5 only. In summary, these periodontal variables showed that e‐cigarette users had the least favorable treatment outcome compared with non‐smokers, followed by the current smokers and former smokers.

To strengthen the study, analysis was undertaken restricting the time to 6‒12 weeks from last PMPR to reassessment (see Figures S1–S3 in online *Journal of Periodontology*). Compared with non‐smokers, e‐cigarette users and smokers still had a higher need for further treatment.

The periodontal treatment response for e‐cigarette users and current smokers was also compared (see Table S12 and Figure S5 in online *Journal of Periodontology*). There were no statistically significant differences in the treatment response between e‐cigarette users and smokers.

When estimating smoking contrasts between e‐cigarette users and former smokers, (see Figure S6 and Table S12 in online *Journal of Periodontology*), outcome levels at re‐evaluation differed significantly between e‐cigarette users and former smokers for the primary outcome (for follow‐up months 6, 7, and 8), the number of sextants with PD ≥5 mm (for months 4, 5, 6, 7, and 8), the number of sites with PD ≥5 mm (for months 6, 7, and 8), mean PD (for months 6, 7, and 8).

To know whether these effects were associated with e‐cigarette use, as opposed to e‐cigarette users also quitting smoking cigarettes in the past, analyses were repeated restricting patients to former smokers and e‐cigarette users (see Figure S7 in online *Journal of Periodontology*). After adjustment for time of smoking cessation, the two groups still differed significantly in their treatment response for the primary outcome (for months 6, 7, and 8), number of sextants with PD ≥5 mm (for months 6, 7, and 8), number of sextants with PD ≥5 mm (for months 7 and 8), and mean PD (for months 6 and 7).

## 4 DISCUSSION

### 4.1 Main findings

The aim of this study was to compare the periodontal treatment response in e‐cigarettes users, current smokers, former smokers, and non‐smokers. All four groups initially presented with similar levels of disease, but post‐PMPR (supra‐ and subgingival) they had statistically significant differences in their levels of disease. Non‐smokers had significantly fewer sites with PD ≥5 mm than the other groups at re‐evaluation, suggesting that they responded to periodontal treatment with the most superior outcome. The e‐cigarette users and the current smokers had the highest “need for surgery” post‐PMPR.

These results demonstrate high clinical meaningfulness. Using this primary outcome, the results showed that post‐PMPR, current smokers and e‐cigarette users needed two extra sextants requiring further treatment in comparison with non‐smokers.

Although we chose the outcome “need for surgery” to be of particular clinical relevance, most previous studies in the literature have used mean PD^26^ and CAL changes as primary outcome variables.^8^, ^9^, ^27^ Therefore, it is worth comparing these results with previous studies using these variables. We can see that from the total population there was a baseline mean PD of 3.33 mm. Post‐PMPR this reduced to 2.65 mm, showing an average reduction of 0.68 mm. This demonstrated that overall, the treatment was successful, resulting in a reduction in PD generally across the entire population. Non‐smokers had a reduction of 0.72 mm mean PD and 0.54 mm CAL gain. This is similar to the current evidence, showing expected PD reduction following PMPR of ≈ 1 mm and 0.5 mm CAL gain.^9^ Suvan et al (2020) demonstrated in a systematic review that the number of closed pockets (PD ≤4 mm and absence of BOP) was 74% after non‐surgical periodontal treatment at 6 months. Our results show the mean number of pocket closure for non‐smokers post‐treatment was 74.9%.^27^

Our results are similar to the current evidence on smoker's treatment response. Jin et al. (2000) showed that 3 months post‐non‐surgical periodontal treatment the mean PD reduction was 1.1 mm in smokers and 2.4 mm non‐smokers, demonstrating non‐smokers responding 2.2 times better than smokers.^8^ Our findings show that smokers had a mean PD reduction of 0.62 mm and non‐smokers had a mean PD reduction of 0.72 mm, demonstrating non‐smokers responding 1.2 times better than smokers. Our study shows that former smokers had a mean PD reduction of 0.72 mm, similar to the response rate of non‐smokers. This reflects the current evidence that the reduction of mean PD for former smokers is comparable with non‐smokers.^28^

There is no current evidence comparing periodontal treatment response in e‐cigarette users to non‐smokers, former smokers, and current smokers, and therefore these results of this study cannot be compared with the existing literature. This study shows statistically significant differences in treatment responses when comparing the non‐smokers group to the e‐cigarette users group. The former smokers, current smokers, and e‐cigarette groups had significantly more sextants with ≥2 non‐adjacent sites of PD ≥5 mm in comparison with non‐smokers, meaning more need for further treatment. From this data the e‐cigarette users responded with the least favorable outcome amongst the groups and were more likely to have sextants with ≥2 non‐adjacent sites of PD ≥5 mm in comparison with the non‐smokers post‐PMPR. The poorer treatment response to PMPR in e‐cigarette users was also significant when analyzing the number of sites with PD ≥5 mm, mean PD, mean CAL, and number of sextants with PD ≥5 mm. There were no significant differences seen between e‐cigarette users and the current smoker groups in this study. However, this result needs to be treated with caution as these two groups were relatively small, and the study was not powered to specifically examine this question.

Wadia et al. (2016) showed an increase in BOP in subjects from switching to e‐cigarette users from smoking cigarettes in a short‐term pilot study of 2 weeks.^13^ This was interpreted as suggesting a reversal of gingival response from a smoker to a non‐smoker. Although these results cannot be directly compared with this study, we have shown no significant difference in FMBS between e‐cigarette users and cigarette users at baseline or re‐evaluation.

### 4.2 Limitations

Some limitations deserve consideration. One of the main limitations of the study was it being retrospective. This can be thought of being inferior in the evidence hierarchy compared with prospective studies. Reasons for this include the risk of selection bias. However, to try and reduce this bias, consecutive subjects were sampled rather than recruiting subjects based on convenience sampling.

Secondly, methodological difficulties can occur especially if unstandardized protocols are followed. For example, there was no formal sample size calculation as there were no data available in the literature on periodontal treatment response to use to estimate the sample size. Therefore, this calculation was based on several assumptions.

Thirdly, the definition for “need for surgery” aims to be clinically relevant, but it is arbitrary and possibly questionable. It was based on the practical consideration that a single 5‐mm residual pocket in one sextant does not necessarily lead to the decision of performing periodontal surgery. However, other periodontal parameters, including at least one site with PD ≥5 mm were used and reported, yielding consistent results.

Fourthly, the operators of the study were 13 periodontal postgraduates. Therefore, there may be inaccuracies of the information recorded as they are not specialized and were not calibrated.

Fifthly, it is important to note that the smoking status of each patient was based on self‐reporting. Although this may introduce inaccuracies due to a risk that patients may underreport their smoking status, studies have demonstrated reliability on the self‐reporting of smoking status.^29^ Future studies may consider measuring smoking status of patients through other means such as cotinine levels. However, cotinine levels may not identify if e‐cigarette users are only using e‐cigarettes or if they are smoking alongside this, as cotinine would also be detected in e‐cigarettes. Perhaps specific metabolites of tobacco, for example, anabasine, could be used instead.^19^ It is also important to recognize the challenges in interpreting the results for this group of patients, as e‐cigarette users are likely to be former smokers. This could be a confounding factor as it could be argued that they are more “hardened smokers” having failed or not wanted to quit using conventional methods of smoking cessation. Previous exposure to smoking may contribute to their treatment responses seen. However, the comparison between e‐cigarette users and former smokers in this study mitigates this limitation. Being retrospective, collection of periodontal indices and patient information could not be checked and scrutinized as it would be in a prospective study, especially not knowing the duration of e‐cigarette use. Additionally, we were unable to determine the previous smoking history in the e‐cigarette group, other than the number of years they had stopped smoking cigarettes. It would have also been beneficial to assess the type of e‐cigarettes used (in terms of flavoring, nicotine content, ratio of propylene glycol, and vegetable glycerin).

Sixthly, due to the nature of this study, we were unable to randomly allocate smoking and e‐cigarettes to the subjects. This may be difficult to control with future studies as it would be unethical to randomly allocate smoking or e‐cigarette use to subjects.

### 4.3 Strengths

A strength of the study is its novelty as, despite extensive resources placed in smoking and outcomes of smoking on periodontal treatment, there is little substantial evidence to verify the effects of using e‐cigarettes on treatment outcome. Although using e‐cigarettes is relatively new, its use is increasing and therefore highlights the importance of understanding its effects on periodontal health.

## 5 CONCLUSIONS

Overall, this study shows that e‐cigarette users responded less favorably than non‐smokers and in a similar fashion to current smokers to PMPR. This resulted in a clinically relevant higher “need for surgery” or at least need for further treatment post‐PMPR.

To our knowledge, this study is the first to assess periodontal treatment response of e‐cigarette users. Therefore, more robust study designs assessing this would be needed to confirm the findings of this exploratory study. There is a need for prospective studies to assess this, although the difficulties with doing so would incur ethical constraints and difficulty in assessing the use of only e‐cigarette dosage without relying on patient self‐reporting. There is some value in exploring the response of e‐cigarettes to periodontal treatment. This information would prove to be useful in advising patients with periodontitis about the effects of e‐cigarettes on their treatment outcome when trying to stop smoking. Currently the advice is that e‐cigarettes may provide support in smoking cessation, but the results it may have on periodontal treatment outcomes are unknown. This study makes one step forward in understanding these effects of e‐cigarettes on periodontal treatment outcomes and encourages more robust studies to confirm these findings.

## CONFLICTS OF INTEREST

There was no funding for the study and there are no commercial or financial relationships of any of the authors.

## AUTHOR CONTRIBUTIONS

C.S., F.J.H. and L.N. made substantial contributions to conception and design of the study. C.S., B.H. and L.N. have been involved in data collection and data analysis. C.S., B.H., F.H.J. and L.N. have been involved in data interpretation, drafting the manuscript and revising it critically and have given final approval of the version to be published.

## Supporting information



Supporting Information

Supporting Information

Supporting Information

Supporting Information

Supporting Information

Supporting Information

Supporting Information

Supporting Information

Supporting Information

Supporting Information

Supporting Information

Supporting Information

Supporting Information

Supporting Information

Supporting Information

Supporting Information

Supporting Information

Supporting Information

Supporting Information

Supporting Information

## Footnotes

* StataCorp (2021). Stata Statistical Software: Release 17. StataCorp, College Station, TX, USA.

† R Core Team (2021). R: A language and environment for statistical computing. R Foundation for Statistical Computing, Vienna, Austria. URL https://www.R‐project.org/
