# Supplementary material for: Retrospective exploratory study of smoking status and e‐cigarette use with response to non‐surgical periodontal therapy
Source: J Periodontol. 2022 Aug 16;94(1):41–54. doi: 10.1002/JPER.21-0702 (PMC10087441; doi:10.1002/JPER.21-0702)
Supplement: Supplementary file 1 — Supporting Information [file JPER-94-41-s002.docx]

Supplementary Table 1: Distribution of operators for smoking groups.

| **OPERATOR** | **NON-SMOKER** | **FORMER SMOKER** | **CURRENT SMOKER** | **E-CIGARETTE USER** |
| --- | --- | --- | --- | --- |
| **1** | 19 | 7 | 2 | 3 |
| **2** | 14 | 6 | 1 | 1 |
| **3** | 14 | 5 | 2 | 3 |
| **4** | 12 | 4 | 1 | 1 |
| **5** | 20 | 4 | 2 | 1 |
| **6** | 30 | 0 | 1 | 1 |
| **7** | 11 | 4 | 2 | 1 |
| **8** | 15 | 3 | 2 | 0 |
| **9** | 14 | 4 | 1 | 1 |
| **10** | 20 | 9 | 0 | 2 |
| **11** | 21 | 8 | 2 | 2 |
| **12** | 18 | 2 | 2 | 2 |
| **13** | 12 | 4 | 2 | 2 |
