# Supplementary material for: Retrospective exploratory study of smoking status and e‐cigarette use with response to non‐surgical periodontal therapy
Source: J Periodontol. 2022 Aug 16;94(1):41–54. doi: 10.1002/JPER.21-0702 (PMC10087441; doi:10.1002/JPER.21-0702)
Supplement: Supplementary file 2 — Supporting Information [file JPER-94-41-s010.docx]

Supplementary Table 2: Results from linear models using generalized least squares for the number of sextants with ≥2 non-adjacent sites of pocket probing depths ≥5 mm.

| **INDEPENDENT VARIABLES** | **B (95% CI)** | **P VALUE** |
| --- | --- | --- |
| Smoking status (ref. non-smokers) |  |  |
| Former smokers | 1.3163 (-1.2017; 3.8343) | 0.3068 |
| Current smokers | 1.4243 (-2.8730; 5.7216) | 0.5167 |
| E-cigarette users | 0.9192 (-3.8400; 5.6783) | 0.7054 |
| RCS1(Treatment duration) (months) | 0.0919 (-0.1792; 0.3630) | 0.5070 |
| RCS2(Treatment duration) (months) | -0.0531 (-0.4857; 0.3795) | 0.8101 |
| Interaction smoking status x treatment duration |  |  |
| Former smokers x RCS1(treatment duration) | -0.1754 (-0.6947; 0.3438) | 0.5086 |
| Current smokers x RCS1(treatment duration) | 0.0074 (-0.8049; 0.8196) | 0.9859 |
| E-cigarette users x RCS1(treatment duration) | 0.1704 (-0.6789; 1.0197) | 0.6946 |
| Former smokers x RCS2(treatment duration) | 0.2297 (-0.5843; 1.0438) | 0.5808 |
| Current smokers x RCS2(treatment duration) | 0.0463 (-1.0362; 1.1287) | 0.9333 |
| E-cigarette users x RCS2(treatment duration) | -0.2950 (-1.4363; 0.8463) | 0.6130 |
| RCS1(Age) (years) | 0.0136 (-0.0330; 0.0601) | 0.5690 |
| RCS2(Age) (years) | -0.0289 (-0.0822; 0.0245) | 0.2905 |
| Male sex | 0.0088 (-0.4562; 0.4737) | 0.9705 |
| Compliant (yes) | 0.1110 (-0.4059; 0.6278) | 0.6744 |
| Number of root surface debridement sessions | 0.5698 (0.2763; 0.8633) | 0.0002 |
| Any medical conditions (yes) | -0.1220 (-0.6148; 0.3708) | 0.6280 |
| Intercept | 0.2331 (-2.0601; 2.5264) | 0.8423 |

Linear regression coefficients (B), 95% confidence intervals (CI) and p values are reported. RCS, restricted cubic spline.
