# Supplementary material for: Retrospective exploratory study of smoking status and e‐cigarette use with response to non‐surgical periodontal therapy
Source: J Periodontol. 2022 Aug 16;94(1):41–54. doi: 10.1002/JPER.21-0702 (PMC10087441; doi:10.1002/JPER.21-0702)
Supplement: Supplementary file 3 — Supporting Information [file JPER-94-41-s007.docx]

Supplementary Table 3: Results from linear models using generalized least squares for the number of sextants with probing pocket depths ≥5 mm.

| **INDEPENDENT VARIABLES** | **B (95% CI)** | **P VALUE** |
| --- | --- | --- |
| Smoking status (ref. non-smokers) |  |  |
| Former smokers | 2.6026 (0.6596; 4.5456) | 0.0093 |
| Current smokers | 0.8789 (-2.4371; 4.1948) | 0.6040 |
| E-cigarette users | 2.0706 (-1.6016; 5.7429) | 0.2704 |
| RCS1(Treatment duration) (months) | 0.0239 (-0.1853; 0.2330) | 0.8233 |
| RCS2(Treatment duration) (months) | -0.0384 (-0.3722; 0.2954) | 0.8218 |
| Interaction smoking status x treatment duration |  |  |
| Former smokers x RCS1(treatment duration) | -0.3821 (-0.7828; 0.0185) | 0.0630 |
| Current smokers x RCS1(treatment duration) | 0.1254 (-0.5013; 0.7522) | 0.6953 |
| E-cigarette users x RCS1(treatment duration) | -0.0658 (-0.7212; 0.5896) | 0.8442 |
| Former smokers x RCS2(treatment duration) | 0.5583 (-0.0698; 1.1864) | 0.0830 |
| Current smokers x RCS2(treatment duration) | -0.1463 (-0.9816; 0.6889) | 0.7317 |
| E-cigarette users x RCS2(treatment duration) | 0.1206 (-0.7600; 1.0013) | 0.7886 |
| RCS1(Age) (years) | -0.0052 (-0.0411; 0.0307) | 0.7766 |
| RCS2(Age) (years) | -0.0043 (-0.0455; 0.0369) | 0.8377 |
| Male sex | -0.2194 (-0.5782; 0.1393) | 0.2320 |
| Compliant (yes) | 0.1441 (-0.2548; 0.5429) | 0.4798 |
| Number of root surface debridement sessions | 0.4430 (0.2166; 0.6695) | 0.0002 |
| Any medical conditions (yes) | -0.3229 (-0.7032; 0.0573) | 0.0976 |
| Intercept | 2.6562 (0.8867; 4.4258) | 0.0036 |

Linear regression coefficients (B), 95% confidence intervals (CI) and p values are reported. RCS, restricted cubic spline.
