# Supplementary material for: Retrospective exploratory study of smoking status and e‐cigarette use with response to non‐surgical periodontal therapy
Source: J Periodontol. 2022 Aug 16;94(1):41–54. doi: 10.1002/JPER.21-0702 (PMC10087441; doi:10.1002/JPER.21-0702)
Supplement: Supplementary file 4 — Supporting Information [file JPER-94-41-s005.docx]

Supplementary Table 4: Results from linear models using generalized least squares for the number of sites with probing pocket depths ≥5 mm.

| **INDEPENDENT VARIABLES** | **B (95% CI)** | **P VALUE** |
| --- | --- | --- |
| Smoking status (ref. non-smokers) |  |  |
| Former smokers | 7.8356 (-15.9602; 31.6314) | 0.5194 |
| Current smokers | -0.5843 (-41.1948; 40.0262) | 0.9775 |
| E-cigarette users | -7.3714 (-52.3461; 37.6034) | 0.7484 |
| RCS1(Treatment duration) (months) | 0.5725 (-1.9894 ;3.1343) | 0.6619 |
| RCS2(Treatment duration) (months) | -0.3880 (-4.4762; 3.7003) | 0.8526 |
| Interaction smoking status x treatment duration |  |  |
| Former smokers x RCS1(treatment duration) | -0.6720 (-5.5789; 4.2350) | 0.7887 |
| Current smokers x RCS1(treatment duration) | 2.1059 (-5.5703; 9.7821) | 0.5914 |
| E-cigarette users x RCS1(treatment duration) | 4.5700 (-3.4564; 12.5963) | 0.2658 |
| Former smokers x RCS2(treatment duration) | 0.9031 (-6.7898; 8.5961) | 0.8182 |
| Current smokers x RCS2(treatment duration) | -2.8679 (-13.0973; 7.3616) | 0.5833 |
| E-cigarette users x RCS2(treatment duration) | -6.1671 (-16.9526; 4.6185) | 0.2638 |
| RCS1(Age) (years) | -0.0673 (-0.5075; 0.3728) | 0.7646 |
| RCS2(Age) (years) | -0.0466 (-0.5509; 0.4577) | 0.8565 |
| Male sex | -0.5809 (-4.9748; 3.8130) | 0.7958 |
| Compliant (yes) | -1.0612 (-5.9457; 3.8233) | 0.6707 |
| Number of root surface debridement sessions | 3.6357 (0.8621; 6.4094) | 0.0109 |
| Any medical conditions (yes) | -4.5768 (-9.2337; 0.0802) | 0.0555 |
| Intercept | 9.8196 (-11.8521; 31.4913) | 0.3756 |

Linear regression coefficients (B), 95% confidence intervals (CI) and p values are reported. RCS, restricted cubic spline.
