# Supplementary material for: Retrospective exploratory study of smoking status and e‐cigarette use with response to non‐surgical periodontal therapy
Source: J Periodontol. 2022 Aug 16;94(1):41–54. doi: 10.1002/JPER.21-0702 (PMC10087441; doi:10.1002/JPER.21-0702)
Supplement: Supplementary file 5 — Supporting Information [file JPER-94-41-s013.docx]

Supplementary Table 5: Results from linear models using generalized least squares for the percentage of pockets with closure.

| **INDEPENDENT VARIABLES** | **B (95% CI)** | **P VALUE** |
| --- | --- | --- |
| Smoking status (ref. non-smokers) |  |  |
| Former smokers | -2.3459 (-27.0762; 22.3844) | 0.8527 |
| Current smokers | 7.2784 (-34.9269; 49.4837) | 0.7357 |
| E-cigarette users | -76.2949 (-123.0357; -29.5540) | 0.0016 |
| RCS1(Treatment duration) (months) | -1.9197 (-4.5821; 0.7428) | 0.1591 |
| RCS2(Treatment duration) (months) | 3.5665 (-0.6823; 7.8153) | 0.1015 |
| Interaction smoking status x treatment duration |  |  |
| Former smokers x RCS1(treatment duration) | -0.2775 (-5.3772; 4.8221) | 0.9152 |
| Current smokers x RCS1(treatment duration) | -2.3904 (-10.3680; 5.5873) | 0.5577 |
| E-cigarette users x RCS1(treatment duration) | 13.8375 (5.4961; 22.1790) | 0.0013 |
| Former smokers x RCS2(treatment duration) | -1.6035 (-9.5985; 6.3916) | 0.6947 |
| Current smokers x RCS2(treatment duration) | 1.4875 (-9.1436; 12.1187) | 0.7842 |
| E-cigarette users x RCS2(treatment duration) | -20.3731 (-31.5822; -9.1640) | 0.0005 |
| RCS1(Age) (years) | 0.1538 (-0.3036; 0.6112) | 0.5106 |
| RCS2(Age) (years) | -0.5918 (-1.1159; -0.0677) | 0.0280 |
| Male sex | -3.2286 (-7.7950; 1.3379) | 0.1674 |
| Compliant (yes) | -3.3180 (-8.3944; 1.7583) | 0.2016 |
| Number of root surface debridement sessions | 2.5109 (-0.3717; 5.3935) | 0.0893 |
| Any medical conditions (yes) | 0.6014 (-4.2384; 5.4412) | 0.8078 |
| Intercept | 158.1664 (135.6436; 180.6891) | <0.0001 |

Linear regression coefficients (B), 95% confidence intervals (CI) and p values are reported. RCS, restricted cubic spline.
