# Supplementary material for: Retrospective exploratory study of smoking status and e‐cigarette use with response to non‐surgical periodontal therapy
Source: J Periodontol. 2022 Aug 16;94(1):41–54. doi: 10.1002/JPER.21-0702 (PMC10087441; doi:10.1002/JPER.21-0702)
Supplement: Supplementary file 6 — Supporting Information [file JPER-94-41-s001.docx]

Supplementary Table 6: Results from linear models using generalized least squares for mean pocket probing depth.

| **INDEPENDENT VARIABLES** | **B (95% CI)** | **P VALUE** |
| --- | --- | --- |
| Smoking status (ref. non-smokers) |  |  |
| Former smokers | 0.6081 (-0.3313; 1.5476) | 0.2060 |
| Current smokers | 0.4896 (-1.1136; 2.0928) | 0.5501 |
| E-cigarette users | 0.8557 (-0.9198; 2.6312) | 0.3460 |
| RCS1(Treatment duration) (months) | 0.0542 (-0.0469; 0.1553) | 0.2949 |
| RCS2(Treatment duration) (months) | -0.0705 (-0.2319; 0.0909) | 0.3929 |
| Interaction smoking status x treatment duration |  |  |
| Former smokers x RCS1(treatment duration) | -0.0833 (-0.2770; 0.1104) | 0.4005 |
| Current smokers x RCS1(treatment duration) | -0.0249 (-0.3280; 0.2781) | 0.8722 |
| E-cigarette users x RCS1(treatment duration) | -0.0425 (-0.3594; 0.2744) | 0.7929 |
| Former smokers x RCS2(treatment duration) | 0.1327 (-0.1710; 0.4364) | 0.3928 |
| Current smokers x RCS2(treatment duration) | 0.0014 (-0.4024; 0.4053) | 0.9945 |
| E-cigarette users x RCS2(treatment duration) | 0.0726 (-0.3532; 0.4984) | 0.7385 |
| RCS1(Age) (years) | -0.0062 (-0.0236; 0.0112) | 0.4840 |
| RCS2(Age) (years) | 0.0051 (-0.0148; 0.0250) | 0.6189 |
| Male sex | -0.0244 (-0.1979; 0.1490) | 0.7827 |
| Compliant (yes) | -0.0080 (-0.2008; 0.1848) | 0.9352 |
| Number of root surface debridement sessions | 0.1673 (0.0578; 0.2768) | 0.0031 |
| Any medical conditions (yes) | -0.1818 (-0.3656; 0.0021) | 0.0540 |
| Intercept | 2.1635 (1.3079; 3.0190) | <0.0001 |

Linear regression coefficients (B), 95% confidence intervals (CI) and p values are reported. RCS, restricted cubic spline.
