# Supplementary material for: Retrospective exploratory study of smoking status and e‐cigarette use with response to non‐surgical periodontal therapy
Source: J Periodontol. 2022 Aug 16;94(1):41–54. doi: 10.1002/JPER.21-0702 (PMC10087441; doi:10.1002/JPER.21-0702)
Supplement: Supplementary file 7 — Supporting Information [file JPER-94-41-s004.docx]

Supplementary Table 7: Results from linear models using generalized least squares for mean recession.

| **INDEPENDENT VARIABLES** | **B (95% CI)** | **P VALUE** |
| --- | --- | --- |
| Smoking status (ref. non-smokers) |  |  |
| Former smokers | 0.3438 (-0.7246; 1.4121) | 0.5290 |
| Current smokers | 1.1956 (-0.6276; 3.0188) | 0.2002 |
| E-cigarette users | 1.0849 (-0.9343; 3.1040) | 0.2936 |
| RCS1(Treatment duration) (months) | 0.0535 (-0.0615; 0.1685) | 0.3631 |
| RCS2(Treatment duration) (months) | -0.0708 (-0.2543; 0.1128) | 0.4507 |
| Interaction smoking status x treatment duration |  |  |
| Former smokers x RCS1(treatment duration) | -0.0681 (-0.2884; 0.1522) | 0.5451 |
| Current smokers x RCS1(treatment duration) | -0.1750 (-0.5197; 0.1696) | 0.3207 |
| E-cigarette users x RCS1(treatment duration) | -0.1824 (-0.5427; 0.1780) | 0.3224 |
| Former smokers x RCS2(treatment duration) | 0.1223 (-0.2230; 0.4677) | 0.4883 |
| Current smokers x RCS2(treatment duration) | 0.1627 (-0.2966; 0.6219) | 0.4883 |
| E-cigarette users x RCS2(treatment duration) | 0.3476 (-0.1366; 0.8318) | 0.1610 |
| RCS1(Age) (years) | 0.0198 (0.0001; 0.0396) | 0.0504 |
| RCS2(Age) (years) | -0.0032 (-0.0258; 0.0195) | 0.7834 |
| Male sex | 0.0808 (-0.1165; 0.2780) | 0.4233 |
| Compliant (yes) | -0.0022 (-0.2215; 0.2171) | 0.9843 |
| Number of root surface debridement sessions | 0.0954 (-0.0292; 0.2199) | 0.1349 |
| Any medical conditions (yes) | -0.0428 (-0.2519; 0.1662) | 0.6885 |
| Intercept | -0.6985 (-1.6715; 0.2744) | 0.1609 |

Linear regression coefficients (B), 95% confidence intervals (CI) and p values are reported. RCS, restricted cubic spline.
