# Supplementary material for: Retrospective exploratory study of smoking status and e‐cigarette use with response to non‐surgical periodontal therapy
Source: J Periodontol. 2022 Aug 16;94(1):41–54. doi: 10.1002/JPER.21-0702 (PMC10087441; doi:10.1002/JPER.21-0702)
Supplement: Supplementary file 8 — Supporting Information [file JPER-94-41-s015.docx]

Supplementary Table 8: Results from linear models using generalized least squares for mean clinical attachment loss.

| **INDEPENDENT VARIABLES** | **B (95% CI)** | **P VALUE** |
| --- | --- | --- |
| Smoking status (ref. non-smokers) |  |  |
| Former smokers | 0.9893 (-0.5424; 2.5210) | 0.2070 |
| Current smokers | 1.4963 (-1.1177; 4.1104) | 0.2632 |
| E-cigarette users | 1.9661 (-0.9289; 4.8610) | 0.1847 |
| RCS1(Treatment duration) (months) | 0.1210 (-0.0439; 0.2859) | 0.1518 |
| RCS2(Treatment duration) (months) | -0.1635 (-0.4266; 0.0997) | 0.2248 |
| Interaction smoking status x treatment duration |  |  |
| Former smokers x RCS1(treatment duration) | -0.1644 (-0.4803; 0.1514) | 0.3088 |
| Current smokers x RCS1(treatment duration) | -0.1598 (-0.6539; 0.3343) | 0.5268 |
| E-cigarette users x RCS1(treatment duration) | -0.2378 (-0.7545; 0.2788) | 0.3680 |
| Former smokers x RCS2(treatment duration) | 0.2766 (-0.2186; 0.7718) | 0.2749 |
| Current smokers x RCS2(treatment duration) | 0.1172 (-0.5413; 0.7756) | 0.7276 |
| E-cigarette users x RCS2(treatment duration) | 0.4418 (-0.2525; 1.1360) | 0.2138 |
| RCS1(Age) (years) | 0.0139 (-0.0144; 0.0423) | 0.3359 |
| RCS2(Age) (years) | 0.0009 (-0.0315; 0.0334) | 0.9547 |
| Male sex | 0.0682 (-0.2147; 0.3510) | 0.6372 |
| Compliant (yes) | -0.0299 (-0.3443; 0.2846) | 0.8525 |
| Number of root surface debridement sessions | 0.2590 (0.0804; 0.4375) | 0.0049 |
| Any medical conditions (yes) | -0.2031 (-0.5029; 0.0967) | 0.1857 |
| Intercept | 1.4268 (0.0318; 2.8218) | 0.0463 |

Linear regression coefficients (B), 95% confidence intervals (CI) and p values are reported. RCS, restricted cubic spline.
