# Supplementary material for: Retrospective exploratory study of smoking status and e‐cigarette use with response to non‐surgical periodontal therapy
Source: J Periodontol. 2022 Aug 16;94(1):41–54. doi: 10.1002/JPER.21-0702 (PMC10087441; doi:10.1002/JPER.21-0702)
Supplement: Supplementary file 9 — Supporting Information [file JPER-94-41-s008.docx]

Supplementary Table 9: Results from linear models using generalized least squares for the full-mouth plaque score.

| **INDEPENDENT VARIABLES** | **B (95% CI)** | **P VALUE** |
| --- | --- | --- |
| Smoking status (ref. non-smokers) |  |  |
| Former smokers | 4.5353 (-17.4171; 26.4877) | 0.6860 |
| Current smokers | -2.9504 (-40.4148; 34.5140) | 0.8775 |
| E-cigarette users | 20.1110 (-21.3795; 61.6015) | 0.3432 |
| RCS1(Treatment duration) (months) | 1.0155 (-1.3478; 3.3789) | 0.4007 |
| RCS2(Treatment duration) (months) | -0.8356 (-4.6071; 2.9360) | 0.6646 |
| Interaction smoking status x treatment duration |  |  |
| Former smokers x RCS1(treatment duration) | -0.4168 (-4.9436; 4.1100) | 0.8570 |
| Current smokers x RCS1(treatment duration) | 1.1565 (-5.9250; 8.2380) | 0.7492 |
| E-cigarette users x RCS1(treatment duration) | -4.1896 (-11.5941; 3.2149) | 0.2688 |
| Former smokers x RCS2(treatment duration) | 1.2117 (-5.8853; 8.3086) | 0.7383 |
| Current smokers x RCS2(treatment duration) | -2.6905 (-12.1275; 6.7464) | 0.5769 |
| E-cigarette users x RCS2(treatment duration) | 7.9295 (-2.0204; 17.8795) | 0.1199 |
| RCS1(Age) (years) | 0.2901 (-0.1159; 0.6961) | 0.1629 |
| RCS2(Age) (years) | -0.2878 (-0.7531; 0.1774) | 0.2267 |
| Male sex | 1.4334 (-2.6200; 5.4869) | 0.4890 |
| Compliant (yes) | -4.7882 (-9.2943; -0.2821) | 0.0385 |
| Number of root surface debridement sessions | -0.2691 (-2.8279; 2.2897) | 0.8369 |
| Any medical conditions (yes) | -2.8734 (-7.1695; 1.4228) | 0.1914 |
| Intercept | 8.1786 (-11.8142; 28.1713) | 0.4236 |

Linear regression coefficients (B), 95% confidence intervals (CI) and p values are reported. RCS, restricted cubic spline.
