# Supplementary material for: Retrospective exploratory study of smoking status and e‐cigarette use with response to non‐surgical periodontal therapy
Source: J Periodontol. 2022 Aug 16;94(1):41–54. doi: 10.1002/JPER.21-0702 (PMC10087441; doi:10.1002/JPER.21-0702)
Supplement: Supplementary file 10 — Supporting Information [file JPER-94-41-s003.docx]

Supplementary Table 10: Results from linear models using generalized least squares for the full-mouth bleeding score.

| **INDEPENDENT VARIABLES** | **B (95% CI)** | **P VALUE** |
| --- | --- | --- |
| Smoking status (ref. non-smokers) |  |  |
| Former smokers | 10.8176 (-11.4564; 33.0915) | 0.3423 |
| Current smokers | -10.8847 (-48.8979; 27.1285) | 0.5853 |
| E-cigarette users | 35.9329 (-6.1654; 78.0312) | 0.0959 |
| RCS1(Treatment duration) (months) | 0.4801 (-1.9179; 2.8781) | 0.6952 |
| RCS2(Treatment duration) (months) | 0.3899 (-3.4369; 4.2167) | 0.8419 |
| Interaction smoking status x treatment duration |  |  |
| Former smokers x RCS1(treatment duration) | -2.1161 (-6.7093; 2.4770) | 0.3676 |
| Current smokers x RCS1(treatment duration) | 2.9052 (-4.2800; 10.0904) | 0.4290 |
| E-cigarette users x RCS1(treatment duration) | -4.9395 (-12.4525; 2.5735) | 0.1990 |
| Former smokers x RCS2(treatment duration) | 1.8895 (-5.3115; 9.09049 | 0.6076 |
| Current smokers x RCS2(treatment duration) | -5.8806 (-15.4558; 3.6946) | 0.2301 |
| E-cigarette users x RCS2(treatment duration) | 4.6691 (-5.4266; 14.7649) | 0.3658 |
| RCS1(Age) (years) | -0.2911 (-0.7031; 0.1209) | 0.1676 |
| RCS2(Age) (years) | 0.2202 (-0.2519; 0.6922) | 0.3617 |
| Male sex | 0.0619 (-4.0509; 4.1748) | 0.9765 |
| Compliant (yes) | -1.6889 (-6.2610; 2.8832) | 0.4699 |
| Number of root surface debridement sessions | 1.3534 (-1.2428; 3.9497) | 0.3081 |
| Any medical conditions (yes) | -1.2559 (-5.6150; 3.1032) | 0.5729 |
| Intercept | 27.7476 (7.4619; 48.0332) | 0.0079 |

Linear regression coefficients (B), 95% confidence intervals (CI) and p values are reported. RCS, restricted cubic spline.
