# Supplementary material for: Retrospective exploratory study of smoking status and e‐cigarette use with response to non‐surgical periodontal therapy
Source: J Periodontol. 2022 Aug 16;94(1):41–54. doi: 10.1002/JPER.21-0702 (PMC10087441; doi:10.1002/JPER.21-0702)
Supplement: Supplementary file 11 — Supporting Information [file JPER-94-41-s017.docx]

Supplementary Table 11: Results from linear models using generalized least squares for the number of teeth (excluding wisdom teeth).

| **INDEPENDENT VARIABLES** | **B (95% CI)** | **P VALUE** |
| --- | --- | --- |
| Smoking status (ref. non-smokers) |  |  |
| Former smokers | 0.3714 (-3.9219; 4.6647) | 0.8655 |
| Current smokers | -2.8900 (-10.2171; 4.4371) | 0.4404 |
| E-cigarette users | 12.6301 (4.5156; 20.7446) | 0.0026 |
| RCS1(Treatment duration) (months) | 0.3485 (-0.1137; 0.8107) | 0.1410 |
| RCS2(Treatment duration) (months) | -0.6313 (-1.3689; 0.1063) | 0.0950 |
| Interaction smoking status x treatment duration |  |  |
| Former smokers x RCS1(treatment duration) | -0.0249 (-0.9102; 0.8604) | 0.9561 |
| Current smokers x RCS1(treatment duration) | 0.5880 (-0.7969; 1.9730) | 0.4063 |
| E-cigarette users x RCS1(treatment duration) | -2.3439 (-3.7920; -0.8958) | 0.0017 |
| Former smokers x RCS2(treatment duration) | 0.3973 (-0.9907; 1.7853) | 0.5754 |
| Current smokers x RCS2(treatment duration) | -0.3892 (-2.2348; 1.4564) | 0.6798 |
| E-cigarette users x RCS2(treatment duration) | 3.3482 (1.4023; 5.2942) | 0.0009 |
| RCS1(Age) (years) | 0.0219 (-0.0575; 0.1013) | 0.5897 |
| RCS2(Age) (years) | 0.0513 (-0.0397; 0.1423) | 0.2703 |
| Male sex | 0.7672 (-0.0256; 1.5599) | 0.0593 |
| Compliant (yes) | 0.1787 (-0.7025; 1.0600) | 0.6914 |
| Number of root surface debridement sessions | -0.4038 (-0.9042; 0.0966) | 0.1153 |
| Any medical conditions (yes) | 0.4332 (-0.4070; 1.2734) | 0.3134 |
| Intercept | -0.3492 (-4.2592; 3.5609) | 0.8612 |

Linear regression coefficients (B), 95% confidence intervals (CI) and p values are reported. RCS, restricted cubic spline.
