# Supplementary material for: Retrospective exploratory study of smoking status and e‐cigarette use with response to non‐surgical periodontal therapy
Source: J Periodontol. 2022 Aug 16;94(1):41–54. doi: 10.1002/JPER.21-0702 (PMC10087441; doi:10.1002/JPER.21-0702)
Supplement: Supplementary file 12 — Supporting Information [file JPER-94-41-s009.docx]

Supplementary Table 12: Estimated contrasts (with 95% confidence intervals and p values) based on linear models from Supplementary Tables 2-11.

|  | **FORMER SMOKERS –**  **NON-SMOKERS** | | **CURRENT SMOKERS –**  **NON-SMOKERS** | | | **E-CIGARETTE USERS –**  **NON-SMOKERS** | | **E-CIGARETTE USERS –**  **CURRENT SMOKERS** | | **E-CIGARETTE USERS –**  **FORMER SMOKERS** | | |
| --- | --- | --- | --- | --- | --- | --- | --- | --- | --- | --- | --- | --- |
| **Treatment**  **duration** | **Contrast (95% CI)** | **P value** | **Contrast (95% CI)** | **P value** | **Contrast (95% CI)** | | **P value** | **Contrast (95% CI)** | **P value** | **Contrast (95% CI)** | **P value** |  |
| **Number of sextants with ≥2 non-adjacent sites of pocket probing depths ≥5 mm** | | | | | | | | | |  |  |  |
| 4 | 0.616 (-0.123; 1.355) | 0.102 | 1.454 (0.125; 2.783) | 0.032 | 1.599 (-0.094; 3.291) | | 0.064 | 0.145 (-1.928; 2.217) | 0.891 | 1.599 (-0.094; 3.291) | 0.064 |  |
| 5 | 0.458 (-0.183; 1.099) | 0.162 | 1.465 (0.524; 2.406) | 0.002 | 1.747 (0.505; 2.988) | | 0.006 | 0.282 (-1.182; 1.746) | 0.706 | 1.747 (0.505; 2.988) | 0.006 |  |
| 6 | 0.336 (-0.412; 1.084) | 0.379 | 1.483 (0.530; 2.436) | 0.002 | 1.849 (0.732; 2.965) | | 0.001 | 0.366 (-0.963; 1.694) | 0.590 | 1.849 (0.732; 2.965) | 0.001 |  |
| 7 | 0.263 (-0.572; 1.098) | 0.537 | 1.511 (0.411; 2.611) | 0.007 | 1.887 (0.745; 3.030) | | 0.001 | 0.377 (-1.049; 1.802) | 0.6046 | 1.887 (0.745; 3.030) | 0.001 |  |
| 8 | 0.233 (0.615; 1.080) | 0.591 | 1.548 (0.351; 2.744) | 0.011 | 1.871 (0.725; 3.018) | | 0.001 | 0.324 (-1.171; 1.819) | 0.671 | 1.871 (0.725; 3.018) | 0.001 |  |
| **Number of sextants with probing pocket depths ≥5 mm** | | | | | | | | | |  |  |  |
| 4 | 1.078 (0.508; 1.648) | 0.0002 | 1.380 (0.354; 2.405) | 0.008 | 1.808 (0.502; 3.114) | | 0.007 | 0.429 (-1.171; 2.028) | 0.599 | 1.808 (0.502; 3.114) | 0.007 |  |
| 5 | 0.738 (0.243; 1.233) | 0.004 | 1.494 (0.768; 2.220) | 0.0001 | 1.752 (0.794; 2.709) | | 0.0003 | 0.258 (-0.872; 1.387) | 0.655 | 1.752 (0.794; 2.709) | 0.0003 |  |
| 6 | 0.486 (-0.091; 1.063) | 0.099 | 1.585 (0.850; 2.321) | <0.0001 | 1.714 (0.852; 2.575) | | 0.0001 | 0.128 (-0.897; 1.154) | 0.806 | 1.714 (0.852; 2.575) | 0.0001 |  |
| 7 | 0.352 (-0.292; 0.997) | 0.284 | 1.646 (0.797; 2.495) | 0.0001 | 1.702 (0.820; 2.584) | | 0.0002 | 0.056 (-1.044; 1.156) | 0.920 | 1.702 (0.820; 2.584) | 0.0002 |  |
| 8 | 0.323 (-0.331; 0.977) | 0.333 | 1.679 (0.755; 2.602) | 0.0004 | 1.712 (0.828; 2.597) | | 0.0001 | 0.034 (-1.120; 1.187) | 0.955 | 1.712 (0.828; 2.597) | 0.0001 |  |
| **Number of sites with probing pocket depths ≥5 mm** | | | | | | | | | |  |  |  |
| 4 | 5.154 (-1.827; 12.135) | 0.148 | 7.819 (-4.743; 20.382) | 0.223 | 10.866 (-5.129; 26.861) | | 0.183 | 3.046 (-16.542; 22.635) | 0.761 | 10.866 (-5.129; 26.861) | 0.183 |  |
| 5 | 4.550 (-1.510; 10.611) | 0.141 | 9.708 (0.816; 18.601) | 0.032 | 14.969 (3.238; 26.701) | | 0.012 | 5.261 (-8.574; 19.096) | 0.456 | 14.969 (3.238; 26.701) | 0.102 |  |
| 6 | 4.088 (-2.979; 11.155) | 0.257 | 11.148 (2.142; 20.155) | 0.015 | 18.107 (7.555; 28.659) | | 0.0008 | 6.959 (-5.596; 19.514) | 0.277 | 18.107 (7.555; 28.659) | 0.0008 |  |
| 7 | 3.819 (-4.075; 11.713) | 0.343 | 11.976 (1.580; 22.371) | 0.024 | 19.928 (9.129; 30.726) | | 0.0003 | 7.952 (-5.517; 21.421) | 0.247 | 19.928 (9.129; 30.726) | 0.0003 |  |
| 8 | 3.718 (-4.292; 11.728) | 0.363 | 12.269 (0.960; 23.579) | 0.034 | 20.600 (9.770; 31.431) | | 0.0002 | 8.331 (-5.797; 22.460) | 0.248 | 20.600 (9.770; 31.431) | 0.0002 |  |
| **Percentage of pockets with closure** | | | | | | | | | |  |  |  |
| 4 | -3.467 (-10.723; 3.788) | 0.349 | -2.273 (-15.329; 10.783) | 0.733 | -21.086 (-37.709; -4.462) | | 0.013 | -18.813 (-39.171; 1.545) | 0.070 | -21.086 (-37.709; -4.462) | 0.013 |  |
| 5 | -3.866 (-10.164; 2.432) | 0.229 | -4.551 (-13.792; 4.691) | 0.335 | -8.789 (-20.981; 3.403) | | 0.158 | -4.238 (-18.616; 10.140) | 0.563 | -8.789 (-20.981; 3.403) | 0.158 |  |
| 6 | -4.516 (-11.861; 2.829) | 0.228 | -6.596 (-15.956; 2.765) | 0.167 | 0.317 (-10.650; 11.283) | | 0.955 | 6.912 (-6.136; 19.961) | 0.299 | 0.317 (-10.650; 11.283) | 0.955 |  |
| 7 | -5.508 (-13.712; 2.696) | 0.188 | -8.323 (-19.127; 2.481) | 0.131 | 5.072 (-6.151; 16.295) | | 0.376 | 13.395 (-0.604; 27.393) | 0.061 | 5.072 (-6.151; 16.295) | 0.376 |  |
| 8 | -6.799 (-15.124; 1.525) | 0.109 | -9.773 (-21.527; 1.980) | 0.103 | 6.035 (-5.221; 17.291) | | 0.293 | 15.808 (1.125; 30.492) | 0.035 | 6.035 (-5.221; 17.291) | 0.293 |  |
| **Mean pocket probing depth** | | | | | | | | | |  |  |  |
| 4 | 0.276 (0.0004; 0.552) | 0.049 | 0.390 (-0.106; 0.886) | 0.123 | 0.686 (0.055; 1.318) | | 0.033 | 0.296 (-0.477; 1.070) | 0.453 | 0.686 (0.055; 1.318) | 0.033 |  |
| 5 | 0.203 (-0.037; 0.442) | 0.097 | 0.365 (0.014; 0.716) | 0.042 | 0.649 (0.186; 1.112) | | 0.006 | 0.284 (-0.262; 0.830) | 0.308 | 0.649 (0.186; 1.112) | 0.006 |  |
| 6 | 0.150 (-0.129; 0.429) | 0.291 | 0.341 (-0.015; 0.696) | 0.061 | 0.624 (0.207; 1.040) | | 0.003 | 0.283 (-0.213; 0.779) | 0.263 | 0.624 (0.207; 1.0401) | 0.003 |  |
| 7 | 0.126 (-0.185; 0.438) | 0.428 | 0.316 (-0.094; 0.727) | 0.131 | 0.613 (0.187; 1.040) | | 0.005 | 0.297 (-0.235; 0.829) | 0.274 | 0.613 (0.187; 1.040) | 0.005 |  |
| 8 | 0.127 (-0.189; 0.443) | 0.432 | 0.292 (-0.154; 0.739) | 0.200 | 0.617 (0.189; 1.044) | | 0.005 | 0.324 (-0.233; 0.882) | 0.254 | 0.617 (0.189; 1.044) | 0.005 |  |
| **Mean recession** | | | | | | | | | |  |  |  |
| 4 | 0.072 (-0.241; 0.386) | 0.652 | 0.497 (-0.067; 1.061) | 0.084 | 0.358 (-0.360; 1.076) | | 0.329 | -0.139 (-1.018; 0.741) | 0.757 | 0.358 (-0.360; 1.076) | 0.329 |  |
| 5 | 0.013 (-0.259; 0.285) | 0.924 | 0.334 (-0.065; 0.733) | 0.101 | 0.202 (-0.325; 0.728) | | 0.453 | -0.132 (-0.753; 0.489) | 0.677 | 0.202 (-0.325; 0.728) | 0.453 |  |
| 6 | -0.026 (-0.344; 0.291) | 0.870 | 0.197 (-0.208; 0.601) | 0.341 | 0.100 (-0.374; 0.574) | | 0.679 | -0.096 (-0.660; 0.467) | 0.738 | 0.100 (-0.374; 0.574) | 0.679 |  |
| 7 | -0.040 (-0.394; 0.314) | 0.825 | 0.094 (-0.373; 0.561) | 0.693 | 0.073 (-0.412; 0.558) | | 0.769 | -0.021 (-0.626; 0.583) | 0.945 | 0.073 (-0.412; 0.558) | 0.769 |  |
| 8 | -0.031 (-0.391; 0.329) | 0.866 | 0.022 (-0.486; 0.529) | 0.933 | 0.110 (-0.376; 0.596) | | 0.657 | 0.088 (-0.546; 0.723) | 0.785 | 0.110 (-0.376; 0.596) | 0.657 |  |
| **Mean clinical attachment loss** | | | | | | | | | |  |  |  |
| 4 | 0.334 (-0.116; 0.783) | 0.146 | 0.858 (0.049; 1.666) | 0.038 | 1.0179 (-0.012; 2.047) | | 0.053 | 0.160 (-1.101; 1.421) | 0.804 | 1.018 (-0.012; 2.047) | 0.053 |  |
| 5 | 0.190 (-0.200; 0.580) | 0.340 | 0.707 (0.134; 1.279) | 0.016 | 0.813 (0.058; 1.569) | | 0.035 | 0.107 (-0.784; 0.997) | 0.815 | 0.813 (0.058; 1.569) | 0.035 |  |
| 6 | 0.090 (-0.365; 0.545) | 0.699 | 0.574 (-0.005; 1.154) | 0.052 | 0.678 (-0.001; 1.357) | | 0.0503 | 0.104 (-0.704; 0.912) | 0.801 | 0.678 (-0.001; 1.357) | 0.0503 |  |
| 7 | 0.049 (-0.459; 0.557) | 0.851 | 0.467 (-0.202; 1.136) | 0.172 | 0.637 (-0.058; 1.332) | | 0.072 | 0.171 (-0.696; 1.038) | 0.700 | 0.637 (-0.058; 1.332) | 0.072 |  |
| 8 | 0.059 (-0.456; 0.575) | 0.822 | 0.381 (-0.347; 1.109) | 0.305 | 0.679 (-0.018; 1.376) | | 0.056 | 0.298 (-0.612; 1.207) | 0.521 | 0.679 (-0.018; 1.376) | 0.056 |  |
| **Full-mouth plaque score** | | | | | | | | | |  |  |  |
| 4 | 2.876 (-3.564; 9.317) | 0.381 | 1.657 (-9.933; 13.246) | 0.779 | 3.407 (-11.349; 18.163) | | 0.651 | 1.750 (-16.321; 19.822) | 0.849 | 3.407 (-11.349; 18.163) | 0.651 |  |
| 5 | 2.551 (-3.040; 8.142) | 0.371 | 2.610 (-5.594; 10.813) | 0.533 | -0.183 (-11.005; 10.640) | | 0.974 | -2.792 (-15.555; 9.971) | 0.668 | -0.183 (-11.005; 10.640) | 0.974 |  |
| 6 | 2.416 (-4.104; 8.936) | 0.468 | 3.141 (-5.168; 11.450) | 0.459 | -2.531 (-12.265; 7.204) | | 0.610 | -5.672 (-17.255; 5.911) | 0.337 | -2.531 (-12.265; 7.204) | 0.610 |  |
| 7 | 2.539 (-4.743; 9.822) | 0.494 | 3.098 (-6.492; 12.689) | 0.527 | -3.185 (-13.147; 6.777) | | 0.531 | -6.284 (-18.709; 6.142) | 0.322 | -3.185 (-13.147; 6.777) | 0.531 |  |
| 8 | 2.888 (-4.502; 10.277) | 0.444 | 2.555 (-7.879; 12.988) | 0.631 | -2.364 (-12.356; 7.628) | | 0.643 | -4.918 (-17.952; 8.115) | 0.460 | -2.364 (-12.355; 7.628) | 0.643 |  |
| **Full-mouth bleeding score** | | | | | | | | | |  |  |  |
| 4 | 2.366 (-4.169; 8.901) | 0.478 | 0.695 (-11.064; 12.455) | 0.908 | 16.207 (1.235; 31.179) | | 0.034 | 15.512 (-2.824; 33.848) | 0.097 | 16.207 (1.235; 31.179) | 0.034 |  |
| 5 | 0.393 (-5.280; 6.066) | 0.892 | 3.156 (-5.168; 11.479) | 0.457 | 11.621 (0.640; 22.602) | | 0.038 | 8.465 (-4.485; 21.415) | 0.200 | 11.621 (0.640; 22.602) | 0.038 |  |
| 6 | -1.284 (-7.900; 5.331) | 0.704 | 4.695 (-3.735; 13.126) | 0.275 | 7.766 (-2.112; 17.643) | | 0.123 | 3.070 (-8.682; 14.823) | 0.609 | 7.766 (-2.112; 17.643) | 0.123 |  |
| 7 | -2.558 (-9.947; 4.831) | 0.497 | 4.979 (-4.752; 14.710) | 0.316 | 4.908 (-5.200; 15.016) | | 0.341 | -0.071 (-12.679; 12.537) | 0.991 | 4.908 (-5.200; 15.016) | 0.341 |  |
| 8 | -3.480 (-10.978; 4.017) | 0.363 | 4.168 (-6.418; 14.754) | 0.440 | 2.919 (-7.219; 13.057) | | 0.573 | -1.249 (-14.474; 11.976) | 0.853 | 2.919 (-7.219; 13.057) | 0.573 |  |
| **Number of teeth (excluding wisdom teeth)** | | | | | | | | | |  |  |  |
| 4 | 0.275 (-0.985; 1.534) | 0.669 | -0.541 (-2.807; 1.726) | 0.640 | 3.278 (0.392; 6.163) | | 0.026 | 3.818 (0.284; 7.352) | 0.034 | 3.278 (0.392; 6.163) | 0.026 |  |
| 5 | 0.280 (-0.814; 1.373) | 0.616 | 0.018 (-1.586; 1.622) | 0.982 | 1.187 ( -0.930; 3.304) | | 0.272 | 1.169 (-1.327; 3.665) | 0.359 | 1.187 (-0.930; 3.304) | 0.272 |  |
| 6 | 0.347 (-0.928; 1.622) | 0.594 | 0.516 (-1.109; 2.141) | 0.534 | -0.379 ( -2.283; 1.525) | | 0.696 | -0.895 (-3.160; 1.370) | 0.439 | -0.379 (-2.283; 1.525) | 0.696 |  |
| 7 | 0.499 (-0.925; 1.924) | 0.492 | 0.930 (-0.945; 2.806) | 0.331 | -1.231 (-3.179; 0.718) | | 0.216 | -2.161 (-4.591; 0.269) | 0.081 | -1.231 (-3.179; 0.718) | 0.216 |  |
| 8 | 0.726 (-0.720; 2.171) | 0.325 | 1.272 ( -0.768; 3.313) | 0.222 | -1.459 (-3.413; 0.495) | | 0.144 | -2.731 (-5.280; -0.182) | 0.036 | -1.459 (-3.413; 0.495) | 0.144 |  |
