# Supplementary material for: Retrospective exploratory study of smoking status and e‐cigarette use with response to non‐surgical periodontal therapy
Source: J Periodontol. 2022 Aug 16;94(1):41–54. doi: 10.1002/JPER.21-0702 (PMC10087441; doi:10.1002/JPER.21-0702)
Supplement: Supplementary file 13 — Supporting Information [file JPER-94-41-s016.docx]

Supplementary Table 13: Results from linear models using generalized least squares estimating effects of time from last PMPR to re-evaluation.

| **PERIODONTAL DATA** |  | **B (95% CI)** |
| --- | --- | --- |
| **Number of sextants with ≥2 non-adjacent sites of PPD ≥5 mm** | Crude | 0.725 (0.322; 1.127)* |
|  | Fully adjusted | 0.216 (-0.200; 0.631) |
| **Number of teeth (excluding wisdom teeth)** | Crude | 0.255 (-0.425; 0.935) |
|  | Fully adjusted | 0.451 (-0.280; 1.181) |
| **Full-mouth plaque score, %** | Crude | 4.908 (1.550; 8.266)* |
|  | Fully adjusted | 3.518 (-0.157; 7.194) |
| **Full-mouth bleeding score, %** | Crude | 2.848 (-0.494; 6.190) |
|  | Fully adjusted | 1.465 (-2.250; 5.180) |
| **Number of sites with PPD ≥5 mm** | Crude | 5.430 (1.679; 9.180)* |
|  | Fully adjusted | 0.952 (-2.980; 4.884) |
| **Mean PPD, mm** | Crude | 0.218 (0.070; 0.365)* |
|  | Fully adjusted | 0.048 (-0.107; 0.203) |
| **Mean recession, mm** | Crude | 0.178 (0.011; 0.344)* |
|  | Fully adjusted | 0.121 (-0.057; 0.298) |
| **Mean CAL, mm** | Crude | 0.406 (0.164; 0.647) * |
|  | Fully adjusted | 0.181 (-0.073; 0.435) |
| **Number of sextants with PPD ≥5 mm** | Crude | 0.347 (0.021; 0.674)* |
|  | Fully adjusted | -0.123 (-0.445; 0.199) |
| **% Pockets with closure** | Crude | -1.181 (-5.129; 2.767) |
|  | Fully adjusted | -2.127 (-6.345; 2.091) |

Models were adjusted for age, sex, compliance, number of PMPR sessions and any medical conditions. Abbreviations: PPD, pocket probing depth; CAL, clinical attachment level.
