# Supplementary material for: Retrospective exploratory study of smoking status and e‐cigarette use with response to non‐surgical periodontal therapy
Source: J Periodontol. 2022 Aug 16;94(1):41–54. doi: 10.1002/JPER.21-0702 (PMC10087441; doi:10.1002/JPER.21-0702)
Supplement: Supplementary file 14 — Supporting Information [file JPER-94-41-s012.docx]

Supplemental Figure 1: Predicted contrasts (from left to right: former smokers, current smokers, e-cigarette users) from linear models analyzing effects of smoking on A) the number of sextants with ≥2 non-adjacent sites of pocket probing depth (PPD) ≥5 mm, B) the number of sextants with pocket probing depth ≥5 mm, C) the number of sites with pocket probing depths ≥5 mm, and D) the percentage of pockets with closure. Patients were restricted to those with 6 to 12 weeks between last PMPR and re-assessment (N=151). Predicted contrasts with 95% confidence intervals are shown. The horizontal dashed line equals a contrast of zero (i.e. no difference).
