# Supplementary material for: Retrospective exploratory study of smoking status and e‐cigarette use with response to non‐surgical periodontal therapy
Source: J Periodontol. 2022 Aug 16;94(1):41–54. doi: 10.1002/JPER.21-0702 (PMC10087441; doi:10.1002/JPER.21-0702)
Supplement: Supplementary file 15 — Supporting Information [file JPER-94-41-s014.docx]

Supplemental Figure 2: Predicted contrasts (from left to right: former smokers, current smokers, e-cigarette users) from linear models analyzing effects of smoking on A) mean pocket probing depth, B) mean recession, and C) mean clinical attachment loss. Patients were restricted to those with 6 to 12 weeks between last PMPR and re-assessment (N=151). Predicted contrasts with 95% confidence intervals are shown. The horizontal dashed line equals a contrast of zero (i.e. no difference).
