# Supplementary material for: Retrospective exploratory study of smoking status and e‐cigarette use with response to non‐surgical periodontal therapy
Source: J Periodontol. 2022 Aug 16;94(1):41–54. doi: 10.1002/JPER.21-0702 (PMC10087441; doi:10.1002/JPER.21-0702)
Supplement: Supplementary file 17 — Supporting Information [file JPER-94-41-s006.docx]

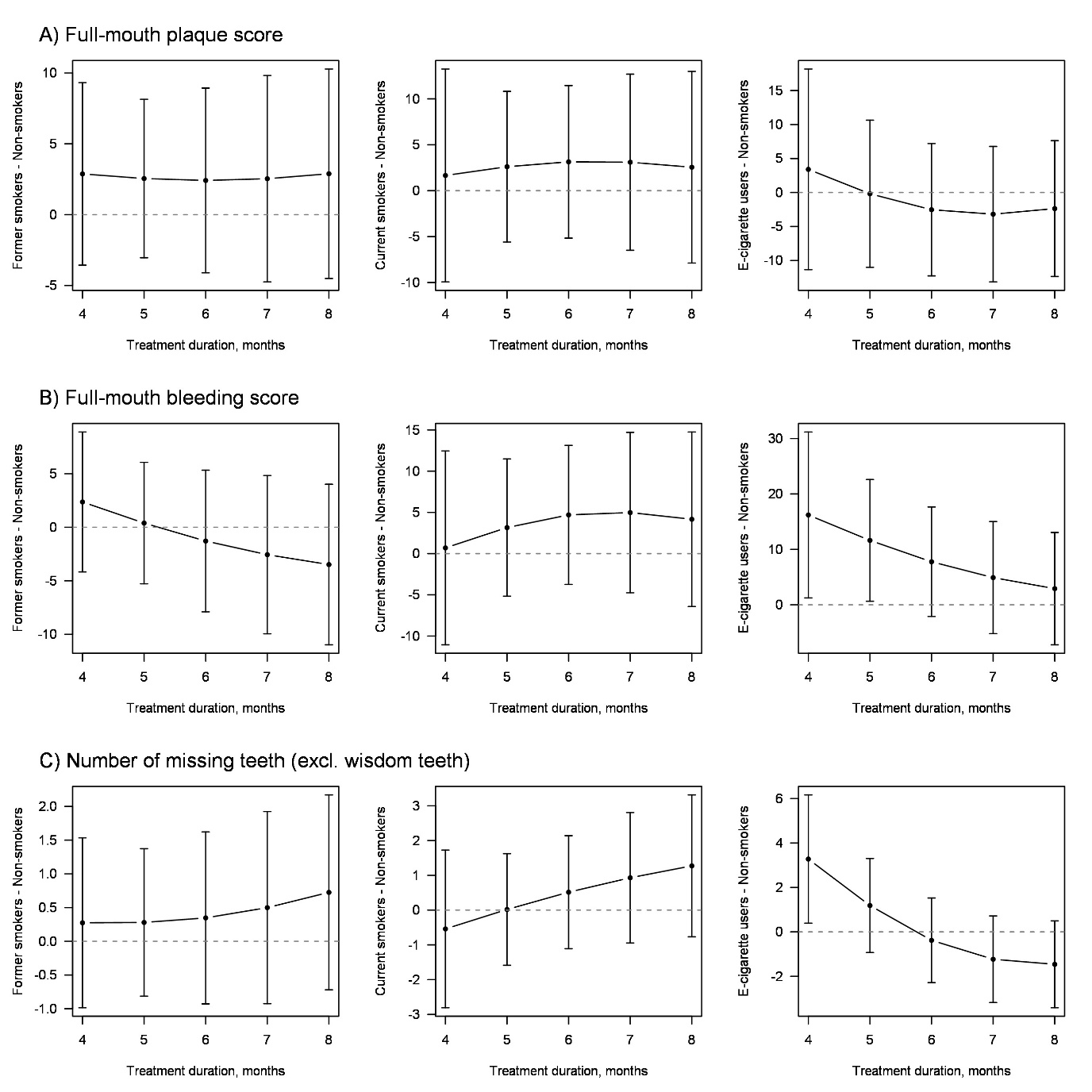


Supplementary Figure 4: Predicted contrasts (from left to right: former smokers, current smokers, e-cigarette users; see data from Supplementary Table 12) from linear models (see Supplementary Tables 9-11) analyzing effects of smoking on A) the full-mouth plaque score, B) the full-mouth bleeding score, and C) the number of missing teeth (excluding wisdom teeth). Predicted contrasts with 95% confidence intervals are shown. The horizontal dashed line equals a contrast of zero (i.e. no difference).
