# Supplementary material for: Retrospective exploratory study of smoking status and e‐cigarette use with response to non‐surgical periodontal therapy
Source: J Periodontol. 2022 Aug 16;94(1):41–54. doi: 10.1002/JPER.21-0702 (PMC10087441; doi:10.1002/JPER.21-0702)
Supplement: Supplementary file 20 — Supporting Information [file JPER-94-41-s011.docx]

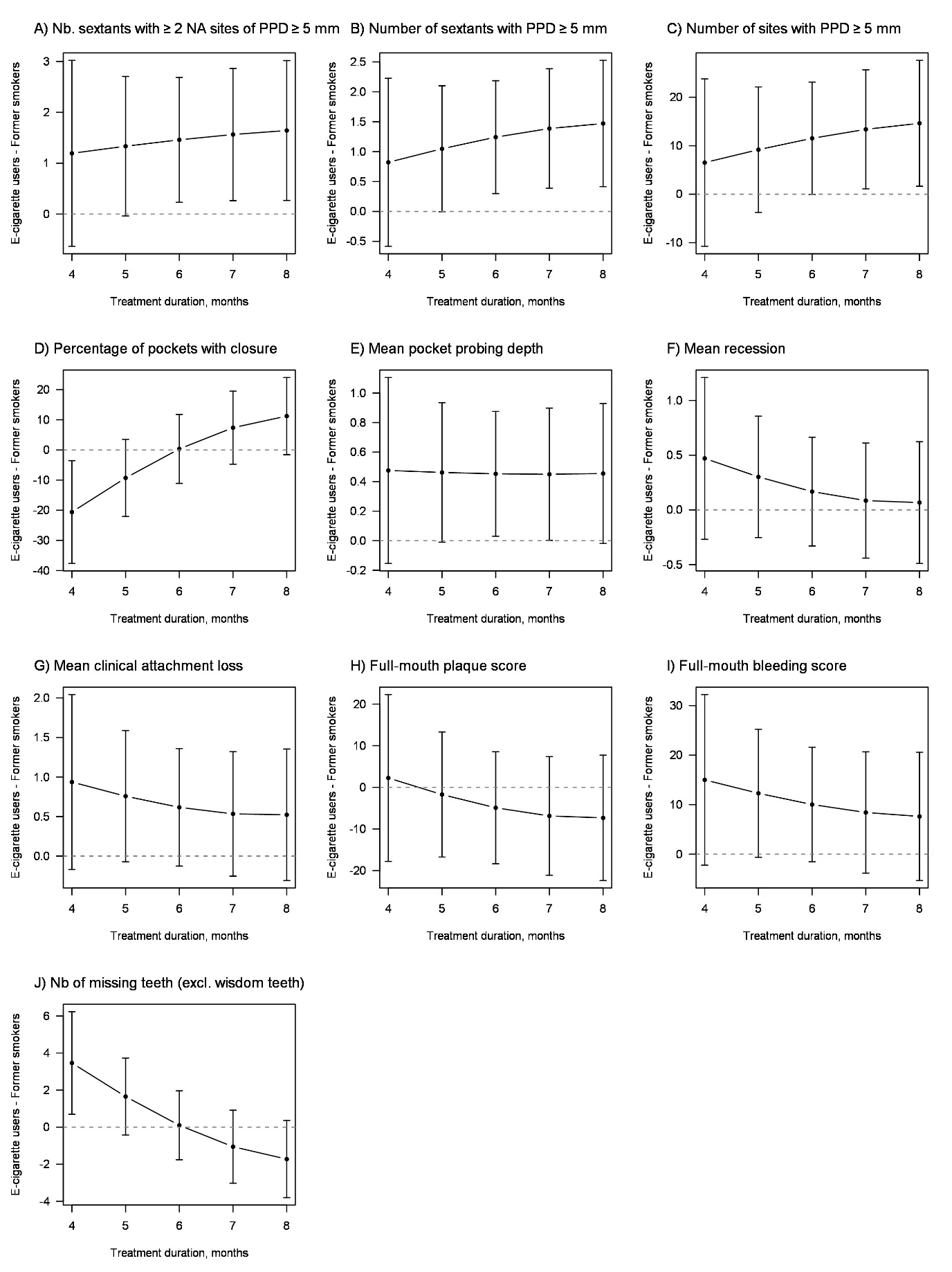


Supplementary Figure 7: Predicted contrasts (e-cigarette users versus former smokers) from linear models analyzing effects of e-cigarette smoking (ref. former smokers) on A) the number (Nb) of sextants with ≥2 non-adjacent (NA) sites of pocket probing depth (PPD) ≥5 mm, B) the number of sextants with pocket probing depth ≥5 mm, C) the number of sites with pocket probing depths ≥5 mm, D) the percentage of pockets with closure, E) mean pocket probing depth, F) mean recession, and G) mean clinical attachment loss, H) the full-mouth plaque score, I) the full-mouth bleeding score, and J) the number of missing teeth (excluding wisdom teeth) with additional adjustment for number of years since quitting. Predicted contrasts with 95% confidence intervals are shown. The horizontal dashed line equals a contrast of zero (i.e. no difference).
